# Atmospheric Pressure Plasma for Carbon Material Modification and Synthesis: A Comprehensive Review

**DOI:** 10.3390/ma18245662

**Published:** 2025-12-17

**Authors:** Siqi Deng, Nozomi Takeuchi, Toshiro Kaneko

**Affiliations:** 1 Graduate School of Engineering, Tohoku University, Sendai 980-8579, Japan; kaneko@tohoku.ac.jp; 2 Department of Electrical and Electronic Engineering, Institute of Science Tokyo, Tokyo 152-8552, Japan; takeuchi@ee.eng.isct.ac.jp

**Keywords:** carbon material modification, atmospheric pressure plasma, carbon material synthesis, plasma reactor design

## Abstract

Atmospheric pressure plasma (APP) has emerged as a versatile tool for the functionalization, modification, and synthesis of carbon-based materials. This review summarizes the historical development, underlying principles, and current progress of APP in material science, with a particular focus on carbon nanomaterials. The fundamentals of plasma parameters are introduced to highlight their roles in driving plasma–surface interactions and establish the diagnostics for these parameters. Recent advances in gas-phase and plasma–liquid systems and the influence of different plasma chemistries have led to different material functionalization results, which are discussed. Applications of plasma-treated carbon in energy storage, environment, and biomedicine are critically reviewed, demonstrating significant improvements in electrochemical performance, adsorption efficiency, and biocompatibility. Finally, current challenges are outlined alongside future perspectives on integrating APP. This review aims to provide a comprehensive reference for researchers seeking to exploit APP as a green and scalable platform for next-generation carbon materials.

## 1. Introduction of APP on Material Science

### 1.1. Atmospheric Pressure Plasma

Plasma is often referred to as the fourth state of matter, distinct from solids, liquids, and gases. It consists of a quasi-neutral mixture of ions, electrons, neutral atoms, and reactive species, generated when a gas is sufficiently energized to cause ionization [1,2]. Due to its unique combination of electrical conductivity, high energy density, and chemical reactivity, plasma has become a powerful tool in various scientific and industrial applications.

**Figure 1:**
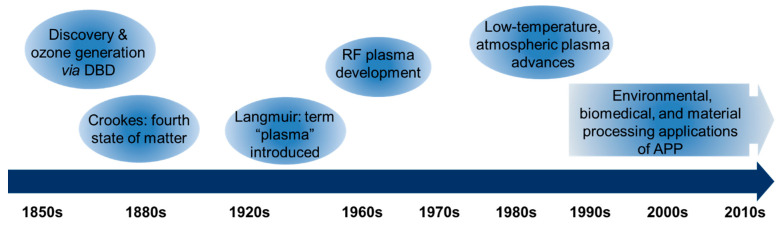
Historical milestones in the development of plasma science.

The study of plasma dates back to the 19th century (Figure 1). In 1855, Theodose du Moncel first reported dielectric barrier discharge (DBD), using planar metal electrodes covered with glass and separated by a narrow gas gap [3,4]. Shortly after, Du Moncel’s discovery was followed by the work of Werner von Siemens, who in 1857 reported on the design and application of a DBD to generate ozone [5]. In 1879, British physicist William Crookes first identified a “fourth state of matter” in low-pressure gas discharges, known as Crookes tubes. In 1928, Irving Langmuir coined the term “plasma” to describe ionized gases that behave collectively, analogous to blood plasma carrying particles, and introduced concepts such as the plasma sheath and electron temperature [1,6]. In the mid-20th century, radio-frequency (RF) plasmas, typically operated near 13.56 MHz, were developed to overcome the limitations of direct current discharges, especially for processing insulating substrates and achieving more uniform treatments [1]. In the late 1980s and early 1990s, engineering advances in the generation of large volume, low-temperature, atmospheric pressure plasma took place [7,8,9,10].

Researchers have worked to make plasma reactors larger at scale. However, at atmospheric pressure, producing diffuse plasmas is inherently challenging. Early efforts therefore employed DBDs, typically using helium as the working gas, to sustain relatively large and uniform plasmas [11]. While the initial motivation was material processing, such as modifying the surface properties of plastics and textiles to make them more hydrophilic or hydrophobic; by the mid-1990s studies revealed an additional application: the effective inactivation of bacteria, gas purification, and water treatment etc. [12,13,14,15,16].

### 1.2. Carbon Materials

Carbon materials (CMs) have attracted tremendous interest due to their unique chemical versatility, structural diversity, and broad applicability across fields such as energy storage, catalysis, electronics, and environmental remediation. The element carbon can form *sp*^1^*, sp*^2^*,* and *sp*^3^ hybridizations, enabling the formation of various allotropes including graphite, diamond, carbon nanotubes (CNTs), fullerenes (C_60_), graphene, and amorphous carbon.

Before 20th century, graphite, carbon black (CB), and activated carbon (AC) were used for centuries. The advent of nanostructured carbon began accelerating in the mid-20th century. Notably, filamentous and tubular carbon structures were reported as early as the 1950s–1970s [17,18,19], although their significance was not fully recognized at the time. In 1985, Harold Kroto et al. discovered buckminsterfullerene which sparked interest in nanoscale carbon structures [20]. In 1991, Iijima reported well-structured multi-walled carbon nanotubes (MWCNTs) in arc-discharge soot [21], a landmark that brought CNTs to the forefront of nanomaterials research and stimulated systematic exploration of their synthesis, properties, and applications. Later, single-walled carbon nanotubes (SWCNTs) were synthesized by arc-discharge soot [22,23]. CNTs quickly became prominent nanoscale materials due to their remarkable physical properties, including high Young’s modulus, ultimate strength and high electric and thermal conductivity [24]. In 2004, Geim and Novoselov demonstrated groundbreaking experiments on two-dimensional graphene using micromechanical exfoliation. Their work provided the first clear route to stable, isolated graphene monolayers and revealed extraordinary electronic and mechanical characteristics intrinsic to single-layer *sp*^2^ carbon [25]. This discovery triggered an explosive expansion of research into 2D carbon systems and related van der Waals materials.

The synthesis of CMs is essential, but to fully exploit their potential in applications, especially at the nanoscale, modification is often required. Many synthesis methods produce materials with limited surface activity or dispersibility. For example, in carbon-based supercapacitors, functionalization can enhance energy storage performance by increasing the accessible surface area, optimizing pore structures, expanding interlayer spacing, and improving electrical conductivity [26,27]. For nanoscale CMs such as CNTs, functionalization typically takes advantage of inherent defect sites, including open ends, sidewall vacancies, pentagon/heptagon irregularities, or oxygen-containing regions. These defects, generated during synthesis or purification, serve as chemically reactive sites. Acid treatments are widely used to stabilize these defect sites by introducing hydroxyl (–OH) and carboxyl (–COOH) groups, mainly at tube ends and occasionally on sidewalls under harsh conditions [28]. These functional groups serve as anchoring points for further chemical modification with moieties such as amines (–NH_2_), amides, esters, and various polymers [29]. An alternative functionalization method involves fluorination of the CNT surface, where elemental fluorine converts *sp*^2^ carbons to *sp*^3^. This disrupts the π-system and lowers conductivity but improves solubility. The attached fluorine atoms can later be substituted with groups such as alkyl, –NH_2_, or –OH, allowing further functionalization.

Broadly, functionalization strategies are divided into covalent and non-covalent approaches [29]. Covalent functionalization involves the formation of covalent chemical bonds between functional groups and the carbon atoms of CNT sidewalls or tips. The method to functionated oxidation, like use HNO_3_, H_2_SO_4_ treatment to introduces –COOH, –OH; amination or esterification; irradiation treatments introducing several kinds of functional groups. This provides strong and durable modification and improves dispersion in polymers, but it partially disrupts the *sp*^2^ lattice, creating defects that may reduce electrical and thermal conductivity [30]. In contrast, non-covalent functionalization relies on weaker interactions, such as π–π stacking, surfactant adsorption, or polymer wrapping, they provide weaker attachment and less control over functional group density compared to covalent approaches [18,31]. These strategies extend across different CMs. Graphene and graphene oxide (GO) can be covalently oxidized or non-covalently modified by aromatic stacking and polymer wrapping [32]. CB and amorphous carbon are oxidized to improve dispersibility or coated with surfactants or polymers [33]. AC undergoes oxidation to enhance adsorption [34], C_60_ can be covalently functionalized or stabilized non-covalently through aromatic or host–guest interactions [20].

### 1.3. Plasma Carbon Modification and Synthesis

Plasma modification has become a powerful tool for controlling the surfaces of CMs. Active species generated in discharges at gas phase or liquid surface, such as electrons, ions, radicals, and metastable, and these active species interact with material surfaces to induce both physical and chemical changes [35]. These processes enable the incorporation of polar oxygen-containing functional groups (OFG), introduce nanoscale roughness, and expose more active sites without altering bulk properties [12,36]. As a result, plasma treatment can optimize electronic structures and impart new functionalities, thereby enhancing catalytic activity, luminescence, adsorption, and other material properties [26,37].

Among plasma techniques with different pressures, APP has attracted particular attention owing to its simplicity, low cost, and scalability. Unlike low-pressure systems, APP operates under ambient conditions without the need for vacuum pumping. Compared with conventional chemical oxidation, APP treatments eliminate solvent use, generate minimal waste, and provide rapid, modification within minutes [38,39]. For CMs, APP offers a clean and tunable alternative to acid oxidation or irradiation. Reactive species such as •O, •OH, NO_x_, and NH_x_ directly graft functional groups (–OH, –COOH, –C=O, –NH_2_) onto surfaces, improving dispersion, interfacial adhesion, and catalytic activity while minimizing over-etching and structural damage [40,41]. Depending on discharge conditions, plasma–material interactions may involve reduction, oxidation, etching, doping, grafting, decomposition, exfoliation, or combined effects [37,40]. Beyond surface functionalization, APP has also been applied to carbon nanomaterial synthesis [37,42,43]. For example, porous carbons were synthesized in situ on metal substrates using a DBD reactor with ethanol vapor as a green precursor [44], and fluorescent carbon quantum dots (CQDs) produced by liquid-phase DBD process react with N,N-dimethylformamide (DMF) as both solvent and carbon source [45].

APP treatment has been widely reported to enhance the mechanical and interfacial performance of CMs. In particular, moderate plasma exposure can increase the tensile, flexural, and impact strengths of carbon fibers and their composites. However, the effect is highly dependent on plasma conditions. Insufficient treatment leads to weak adhesion and poor performance, whereas excessive treatment can cause structural damage and reduce strength. To achieve reliable modification, precise control of discharge parameters is required to balance chemical activation with structural preservation. For optimizing the efficiency and scalability of plasma-based carbon surface modification and synthesis processes, it is essential to understand the key plasma parameters and their relationship with CMs (shown in Table 1).

One of the most important parameters influent plasma behaviors is the electric field in the discharge. It is commonly expressed as an absolute field strength (V m^−1^) or as a reduced electric field (*E*/*N*, in Townsend; 1 Td = 10^−21^ V m^2^, where *N* (m^−3^) denotes neutral gas number density. It characterizes how much energy electrons can gain *per unit distance between collisions*, considering how often they collide with neutral particles. Because in low-temperature plasmas, electrons gain energy from the field but lose it through collisions with neutral molecules. Where higher reduced electric field, electrons can gain more energy between collisions. There are many non-invasive optical diagnostics for electric field strength measurements within the discharge itself. Although electrical measurements could in principle provide information about the internal field, they are difficult to perform reliably in transient, nanosecond, or fast-pulsed discharges. D-dot and B-dot sensors have been employed to characterize nanosecond pulsed HV sources [46], and capacitive probes enable millimeter-scale mapping of potential distributions along the discharge gap [47]. These diagnostic techniques have been widely applied to investigate breakdown front propagation and field evolution in nanosecond discharges, particularly under low-pressure conditions. For many discharges, optical emission spectroscopy (OES) remains the most widely used non-intrusive technique to determine the electric field with high spatial and temporal resolution. OES-based approaches rely either on Stark-effect broadening [48,49] or on collisional–radiative modeling of selected spectral lines [50]. The achievable time resolution is set by the detector, like ICCD, streak cameras, or fast PMTs, generally provide sufficient temporal response for nanosecond plasmas [50]. OES methods usually require plasma emission, and their interpretation relies on specific excitation schemes that must be supported by collisional–radiative modeling. Although most applications have focused on air or helium/hydrogen plasmas [51,52], in principle these techniques can be extended to other gases if two radiative states with different energy thresholds are directly excited. In contrast, laser-based diagnostics can measure electric fields even without plasma emission, allowing information to be obtained before or after a discharge. These methods include laser induced fluorescence dip spectroscopy (LIF-DIP), electric field induced coherent Raman scattering (E-CARS), and electric field induced second harmonic generation (E-FISH). In a typical LIF experiment, a laser tuned to a molecular transition excites the target species in a sheet or line. The subsequent fluorescence is detected by an ICCD camera or a PMT [53,54]. E-CARS works like standard CARS, where pump and Stokes photons excite molecules. The external electric field creates a net molecular dipole, breaking the natural symmetry of the molecules. This makes normally IR-inactive molecules emit IR radiation. The intensity of this radiation depends on the square of the electric field, so by calibrating with a known field, the local electric field of discharge can be measured [55]. LIF-DIP spectroscopy and E-CARS are restricted to certain gases or mixtures, while E-FISH has emerged over the last decade as a powerful and versatile technique which is first operate in plasma research in 2017 [56]. In E-FISH experiment, a laser beam passes through the discharge region, where the electric field breaks molecular symmetry and generates a second harmonic (double frequency) light signal [57,58]. It is relatively simple, compatible with many laser systems, and applicable to virtually any gas. For these reasons, E-FISH is likely to become a standard diagnostic in the coming years in electric field measurements [51].

In plasma research, especially for low-temperature or non-equilibrium plasmas, the behavior of electrons plays a central role in determining plasma properties and chemical reactivity. Two important descriptors are the electron temperature (*T_e_*) and the electron energy distribution function (EEDF). *T_e_* represents the mean kinetic energy of the electron population. The electron energy distribution function, *f*(*ε*), describes the probability density of electrons possessing a given energy *ε* within the plasma. *T_e_* could controls radical generation, which is important in plasma applications, where higher *T_e_* accelerates functional group incorporation. Due to these processes are highly sensitive to electron energy, the shape of the EEDF determines how electron-driven processes occur, such as excitation, ionization, dissociation, and attachment. Various approaches can be used to describe the EEDF, such as using an analytic function like a Maxwellian or Druyvesteyn function or solving the Boltzmann equation [59]. The presence of a high-energy tail in the EEDF is particularly important for processes requiring elevated electron energies, such as the generation of reactive radicals or ionization of molecular gases. EEDF can be obtained either by solving the Boltzmann equation [51] or experimental techniques such as probe diagnostics (main use in low pressure plasma) [60], Thomson scattering [61] or the emission line method [62,63].

The gas temperature (*T_g_*) is the average kinetic energy of neutral gas molecules in the plasma, which refers to the average translational energy of the neutral atoms and molecules. *T_g_* could influence reaction thermodynamics, diffusion rates, surface heating or damage on the material in plasma application; as such, it is important to control it. Direct measurement of *T_g_* in plasmas with a thermometer is difficult, but several accurate indirect methods are available. These techniques include rotational distributions of diatomic molecules, spectral line profile analysis, Rayleigh scattering, and thermal probes [64]. Among these, extracting rotational temperatures from molecular emission or absorption spectra is often considered a reliable indicator of gas temperature, as their lifetimes exceed the mean collision time. Accurate determination of these distributions requires active diagnostics such as LIF [65], absorption spectroscopy [66], or Raman scattering [67]. Atomic and molecular transitions produce emission or absorption features at characteristic wavelengths, but the lines exhibit finite width. Several mechanisms contribute to this broadening, including the intrinsic lifetime of the excited state (natural broadening), the thermal motion of particles (Doppler broadening), and field-induced or collisional effects such as Stark, van der Waals, and pressure broadening [68]. Neutral gas density can be determined most directly through Rayleigh scattering, which measures the elastic scattering of light by bound electrons in atoms and molecules. Under ideal-gas conditions and fixed pressure, this implies that the Rayleigh-scattered intensity varies inversely with the gas temperature [69].

**Table 1:** Key parameters of atmospheric pressure plasma: measurement methods, and relevance to material treatment [70].

Parameter	Definition	Measurement Methods	Relevance to Treated Carbon Performance
Electron temperature (*T_e_*)	Average kinetic energy of electrons, determines excitation/ionization rates	OES, Thomson Scattering	Controls radical generation (O, N, OH); higher *T_e_* accelerates functional group incorporation
Gas temperature (*T_g_*)	Average kinetic energy of neutral species (atoms/molecules)	Rotational spectroscopy (LIF, absorption spectroscopy), spectral line profile analysis (Doppler broadening and van der Waals broadening), neutral density measurements (Rayleigh scattering), and thermal probes	Determines material compatibility; low *T_g_* enables carbon functionalization without structural damage
Electron density (*n_e_*)	Number density of free electrons in plasma	Stark broadening, microwave interferometry, Thomson scattering	Determines production of specific radicals
Electron Energy Distribution Function (EEDF)	Probability distribution of electron energies	Probe diagnostics, Thomson scattering, emission line method	Determines production of specific radicals (e.g., OH, NO_x_, NH_2_)
Reduced Electric Field (*E*/*N*)	Ratio of electric field strength (E) to gas number density (N), in Townsend (Td)	Simulation, inferred from OES, discharge modeling	Governs energy transfer per collision; higher E/N promotes dissociation/ionization
Plasma power or energy	Energy delivered per unit volume	Electrical diagnostics (V–I waveforms), charge-voltage Lissajous curve	Determines process throughput and efficiency of functionalization

Based on the thermodynamic equilibrium, plasma could be divided into low-temperature plasmas (LTPs) and thermal plasma. The majority of LTPs deviate from thermodynamic equilibrium, with the *T_e_* being much higher than the heavy particle and *T_g_*. LTPs are partially ionized gases in which electrons possess high kinetic energies, while ions and neutral molecules remain near ambient temperature. Such plasmas can generate environments rich in reactive species at close to room temperature, under atmospheric pressures. This unique non-equilibrium condition enables the delivery of highly reactive plasma species in a non-destructive manner, making them particularly advantageous for processing heat-sensitive surfaces. Consequently, LTPs are ideal for the selective functionalization of delicate carbon nanomaterials without damaging their structures. In contrast, gliding arc and thermal arc plasmas operate at much higher gas temperatures, which are suitable for large-scale reforming or synthesis processes but less appropriate for the treatment of sensitive nanostructured materials. Common plasma sources and their parameter are shown in Table 2.

In addition to plasma diagnostic techniques, a comprehensive understanding of plasma-induced modifications requires appropriate analytical tools capable of probing structural changes, defect formation, surface chemistry, and functional group incorporation. Commonly employed methods include BET surface area analysis, residual gas analysis (RGA), and titration-based quantification of acidic or nitrogen-containing groups. Among these, Raman spectroscopy, infrared (IR) spectroscopy, and X-ray photoelectron spectroscopy (XPS) are particularly powerful for characterizing CNTs, graphene, and related carbon nanomaterials due to their sensitivity to bonding configurations and vibrational or electronic structures.

Raman spectroscopy is one of the most widely used techniques to assess structural disorder and graphitic quality after plasma treatment. Key Raman features G band which located around 1580 cm^−1^ corresponds to the E_2_g phonon mode of *sp^2^*-bonded carbon. Its position and full width at half maximum (FWHM) are indicators of graphitic order. The D band (~1350 cm^−1^), activated by defects, increases in intensity as plasma treatments introduce vacancies, edge sites, and functional groups. The commonly used *I_D_*/*I_G_* ratio provides a quantitative measure of defect density. For example, in DBD air plasma *I_D_*/*I_G_* ratio showed that plasma did not disrupt graphitic structure, indicating controlled surface modification [72]. The 2D band (~2680 cm^−1^) is highly sensitive to layer number and stacking order in graphene; plasma-induced etching or doping typically reduces its intensity or alters its line shape. Raman mapping further enables assessment of spatial uniformity, particularly important for atmospheric-pressure plasma systems where discharge distribution may vary locally.

FTIR provides direct evidence of newly introduced functional groups. Main OFGs produced by plasma oxidation are located at: Hydroxyl groups located around 3400 cm^−1^ according to O–H stretching; Carbonyl groups located around 1650–1740 cm^−1^ according to C=O stretching; Carboxyl and lactone groups located around 1200–1300 cm^−1^ according to C–O stretching [73,74,75,76,77,78]. For nitrogen-containing plasmas, characteristic peaks include N–H stretching: 3200–3400 cm^−1^; C–N or C=N vibrations: 1200–1600 cm^−1^ [79,80]. In plasma–liquid systems, FTIR clearly identifies sulfur-containing groups such as –SO_3_H (1040–1180 cm^−1^) when H_2_SO_4_ or sulfate precursors are used [81,82], where strong SO stretching bands confirm covalent sulfonation of CNTs and related materials.

XPS provides quantitative information on elemental composition and chemical bonding states and is widely used to confirm and quantify O-, N-, and S-containing functional groups on plasma-treated carbon surfaces [78,83,84,85,86]. Because XPS probes only the top few nanometers, its results should be interpreted alongside titration-based measurements to assess the true functional group density. Misinterpretation of fitted peaks is common in the literature; therefore, careful and consistent curve fitting is essential. Peak positions and recommended full widths at half maximum (FWHM) follow values compiled in the XPS Handbook by John F. Moulder [87]. C 1s calibration at ~284.5 eV (C=C) is the first step, followed by deconvolution into:), C–C and C–H located at 284.8–285.0 eV, C–OH, C–O–C located at 286.3–286.7 eV, C=O located at 287.8–288.0 eV; O–C=O C=O located at 289.0–289.3 eV [88,89,90,91]. These fits typically employ FWHM values of 0.9–1.3 eV for oxygenated components, while the graphitic C=C peak is narrower (0.4–0.8 eV). Fitting C 1s rather than O 1s is generally preferred because O 1s frequently contains overlapping contributions from multiple oxygen-containing species.

Other complementary analytical techniques further strengthen the characterization framework. TEM and SEM visualize morphological changes, defect formation, and plasma-induced etching. TGA assesses the thermal stability of plasma-generated functional groups. Contact angle measurements and BET surface area analysis provide indirect evidence of surface oxygenation, wettability, and porosity modifications [92,93,94]. Together, these methods allow correlation between plasma operating conditions and the resulting changes in carbon surface chemistry, crystallinity, defect distribution, and functional group incorporation.

Although APP methods have shown remarkable promise for the synthesis and surface modification of carbon materials, the literature is highly fragmented. Most studies focus on individual plasma configurations, specific gas chemistries, or narrow material classes, making it difficult for researchers and industry practitioners to obtain a unified understanding of how different APP designs, discharge conditions, and substrate properties influence carbon material formation and functionalization. In addition, most existing review articles on plasma-based material processing are centered on low-pressure plasma technologies, whose underlying physics, reactor architectures, and processing regimes differ substantially from those of APP systems. Consequently, a systematic and consolidated overview dedicated specifically to atmospheric-pressure plasma processing is still lacking. The motivation of this review is to bridge this gap by organizing the scattered knowledge into a coherent framework that highlights fundamental mechanisms, reactor architectures, functionalization behavior, and energy-efficiency considerations across the major categories of APP systems. This work is intended for a broad audience who seek an accessible yet comprehensive guide to APP-based carbon material processing. By integrating insights from plasma science, materials chemistry, and engineering practice, this review aims to provide a foundational platform for future research, technology development, and industrial implementation of APP methodologies.

## 2. Design of APP Reactor on Material Treatment

The design of an APP reactor plays a decisive role in determining the efficiency, selectivity, and scalability of material treatment. Unlike low-pressure plasmas, APP systems operate without vacuum equipment, making them more attractive for industrial applications. However, this advantage introduces challenges in controlling discharge uniformity, plasma stability, and heat management. This chapter provides an overview of common APP reactor configurations, followed by an outlook on reactor design trends, emphasizing strategies for achieving large-area uniformity, energy efficiency, and process scalability.

### 2.1. Common APP Reactor Configurations

Several reactor configurations have been developed for APP-based material treatment, each exhibiting distinct discharge characteristics and application scopes. Depending on the interaction mode between plasma and matter, APP systems can be broadly classified into four categories: gas-phase plasmas, plasmas above liquid, in-liquid plasmas, and hybrid/advanced systems. Each category has its own advantages, limitations, and suitable reactor designs, which are summarized in Table 3 and further discussed in the following sections.

**(a) Gas-phase plasmas**

*Dielectric Barrier Discharge (DBD) Reactors*

DBDs are the most widely used configuration for material processing [4]. A DBD reactor consists of two electrodes separated by at least one dielectric layer, which is usually made of quartz or ceramic. Generally, the discharge usually forms as numerous transient microfilaments driven by AC or pulsed DC at atmospheric pressure,, with operating frequencies ranging from tens of Hz to several tens of kHz [13].

**Figure 2:**
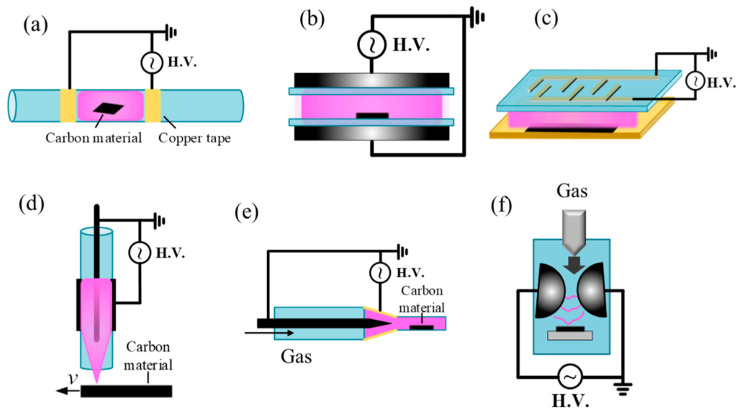
Schematic of several types of gas-phase plasma setups for material science; (**a**) coaxial type DBD reactor; (**b**) plate-to-plate DBD reactor; (**c**) surface–DBD systems; (**d**,**e**) APPJ; (**f**) gliding arc reactors.

DBD reactors are generally classified into two geometries: plate-to-plate and coaxial. A common coaxial design of plasma reactor for carbon treatment is shown in Figure 2a. This design employs a quartz tube wrapped with a metal coil or plate, ensuring that both the working gas and the processed material remain isolated from the electrodes. This configuration effectively prevents undesired electrode contamination on the treated carbon. Depending on the requirements of the catalytic materials to be processed, carbon samples may be exposed directly within the discharge zone or downstream of the plasma. Since plasma treatment primarily affects surfaces, powders require mechanical or manual stirring to ensure uniform exposure. To solve this issue, some researchers have developed 360° rotating plasma systems, which have been applied for the preparation of supported metal catalysts [95]. Compared with the coaxial designs, plate-to-plate reactors are easier to scale up for large-area processing (Figure 2b). However, coaxial systems are often more effective in sustaining discharges due to their geometry. Thanks to their scalability and compatibility with roll-to-roll processing, DBDs are now widely employed for material modification, enabling uniform, non-equilibrium surface functionalization [37]. Applications include surface activation, oxidation [96,97], and heteroatom doping of CMs. Nonetheless, filamentary discharges in certain gases can compromise uniformity, necessitating careful optimization of parameters such as electrode gap, dielectric thickness, and power waveform [98].

DBDs can also be categorized into volume–DBD and surface–DBD systems. In volume–DBDs, micro-discharges occur between the electrodes, whereas in surface–DBDs they develop on the dielectric surface [12]. For catalytic materials, particle forms are often preferred in order to avoid gas trapping. As shown in Figure 2c, the samples are positioned beneath the discharge rather than directly within it. This arrangement is particularly suitable for processing thermally sensitive materials, allowing treatment of particles, or powders type materials without direct exposure to the plasma [99]. Moreover, there are no strict requirements on the dielectric constant, thickness, or conductivity of the processed samples, providing greater flexibility in material selection [37].

*Atmospheric Pressure Plasma Jets (APPJs)*

As shown in Figure 2d,e, a carrier gas is passed through a discharge region between electrodes powered by high-voltage AC, DC, RF, or microwave power sources. The ionized gas exits the nozzle as a plasma plume containing reactive oxygen and nitrogen species (RONS), UV photons, electrons, and ions, which interact with the substrate [100,101,102]. Because the distance between the plasma jet and the substrate can be precisely controlled, APPJs minimize direct ion bombardment and thermal load, making them particularly suitable for delicate or heat-sensitive materials. APPJs may be configured as single-electrode or double-electrode systems, with or without precursor injection. The key advantages of APPJs include High directionality and localized treatment, compatibility with complex geometries and 3D surfaces, flexibility for targeted surface modifications. Reported applications include functionalization of graphene flakes; localized doping of CNTs; Thin-film deposition of oxides, carons, or metals [103]; improves adhesion, bond strength, and catalytic surface properties; and wettability control [74,104,105]. The main limitations of APPJs are the relatively small treatment area, potential overheating, and non-uniform modifications. For large-scale applications, multi-jet arrays are typically employed to improve throughput and uniformity [106].

*Gliding arc reactors*

Gliding arc plasmas operate in the transitional regime between thermal and non-thermal plasmas, often referred to as warm plasmas. They are generated between diverging electrodes in the presence of a flowing gas, where the arc is stretched and simultaneously cooled by the gas flow (Figure 2f) [107]. This configuration enables stable operation at atmospheric pressure while maintaining a highly reactive environment. The main advantages of gliding arc plasmas include their high density of reactive species and suitability for bulk gas-phase reactions. CMs can either be directly introduced into the plasma zone by nozzle for treatment or positioned downstream, where they are exposed to extended streamers. Reported applications include the synthesis of carbon nanomaterials, large-scale modification of ACs, and plasma-assisted reforming processes [108,109,110,111]. However, compared with DBDs and APPJs, the higher gas temperatures in gliding arc plasmas may pose risks for the structural integrity of delicate nanomaterials [112].

**(b) Plasma-above-liquid & in-liquid plasma reactors**

Discharges can also be generated directly within liquids or at liquid surfaces, producing solvated radicals in the liquid phase along with UV photons. Typical configurations include pin-to-plate discharges in liquid, bubble-assisted discharges [15], and solution plasma processing [71,113]. These systems enable direct functionalization of suspended nanomaterials, facilitating simultaneous oxidation, doping, or nanoparticle synthesis. Despite these advantages, several challenges remain, including electrode erosion, solvent degradation, and limited reproducibility during scale-up.

While plasma–liquid interactions for CMs treatment are still under development, they have been extensively investigated in the field of water treatment over the past decades. In water purification, plasmas are used to generate reactive species capable of decomposing organic contaminants [16,114,115]. The fundamental requirement in both cases is the same: maximizing the plasma–liquid interfacial area to enhance radical production and ensure effective contact with the target [116]. Consequently, reactor design strategies established for plasma-based water treatment may offer valuable insights for advancing plasma–liquid systems in the functionalization of CMs.

*Plasma above liquid*

**Figure 3:**
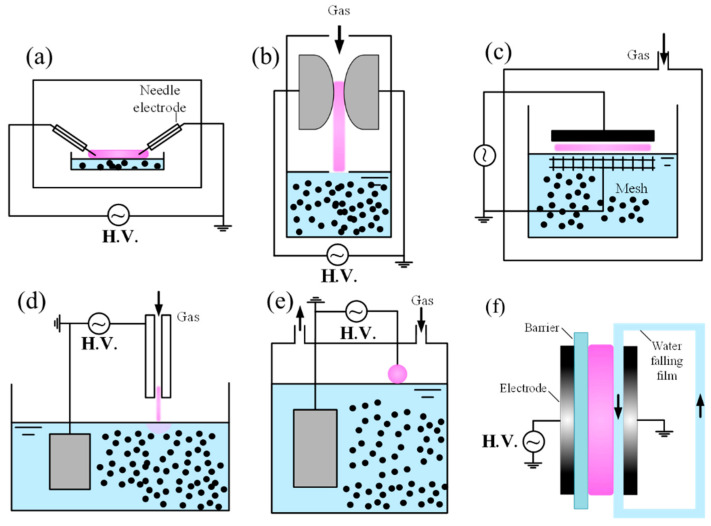
Schematic of several types of plasma-above-liquid setups for material science: (**a**) surface discharge; (**b**) gliding arc reactor; (**c**) DBD reactor; (**d**) APPJ; (**e**) pin-to-liquid reactor; (**f**) DBD with water film.

Similarly to gas-phase plasmas, applied area of plasma discharges with liquid surfaces can also be scaled up by adopting DBD designs (Figure 3c). In such systems, the upper electrode may incorporate a dielectric layer, or alternatively, the liquid itself can serve as the dielectric barrier, like use purified water or other low conductivity organic liquid. This configuration enables uniform discharge between a glass plate and the liquid surface. Importantly, the liquid electrode continuously evaporates water vapor, thereby supplying additional OH radicals, at higher levels than when only H_2_O vapor is introduced into the discharge [96,97]. Although diagnostics for active species density measurement remain limited in plasma–liquid interaction, •OH densities exceeding 10^23^ m^−3^ have been reported [117], which is higher than plasma discharge in water vapor (~10^20^ m^−3^) [118]. Modeling predicts high concentrations of NO, O•, •OH, and HO_2_• [119,120,121,122]. Instabilities at the plasma–liquid interface, driven by strong sheath fields, are thought to enhance gas–liquid mass transfer [123,124]. Furthermore, the liquid acts as a thermal sink, reducing gas temperature when plasma simultaneously contacts the liquid and the CM. This cooling effect helps prevent damage to delicate carbon nanostructures during processing.

Discharges using liquid water electrodes have been investigated in several geometries, including pin-to-liquid and falling-film electrode configurations. The vapor layer formed by electrolysis and evaporation surrounds the liquid electrode under high current conditions, influencing discharge stability [125,126]. Liquid electrodes act similarly to resistive electrodes, helping to stabilize diffuse glow discharges and prevent contraction, even under DC excitation at atmospheric pressure. These systems couple strongly with the liquid phase, with a substantial fraction of the input power dissipated into evaporation. The effect is particularly pronounced when the liquid serves as the cathode. Notably, plasmas sustained with liquid cathodes and liquid anodes exhibit different discharge characteristics, interaction dynamics and even chemical reactions. To address the problem of liquid surface instability, a flowing liquid can be employed to reduce deformation at the plasma–liquid interface. As illustrated in Figure 3f, there are many designs of the plasma’s contact with the water film. However, maintaining a flat and smooth liquid film remains challenging due to fluctuations caused by the water pump and the inherent instabilities of fluid dynamics.

*In-liquid plasma*

Discharges generated directly in liquids are highly dynamic and transient, typically initiated by strong electric fields, bubble implosions, or laser pulses. Non-equilibrium plasmas formed by high-voltage pulses are commonly referred to as streamer or corona discharges, often produced in pin-to-pin or pin-to-plate geometries [127,128]. Microsecond pulsed discharges are generally enabled by pre-existing or voltage-induced bubbles, whereas nanosecond pulses can produce breakdown without bubble formation, likely involving field enhancement at nanopores or near electrodes [71]. Despite extensive study, no comprehensive theory fully explains liquid breakdown, particularly at nanosecond timescales.

**Figure 4:**
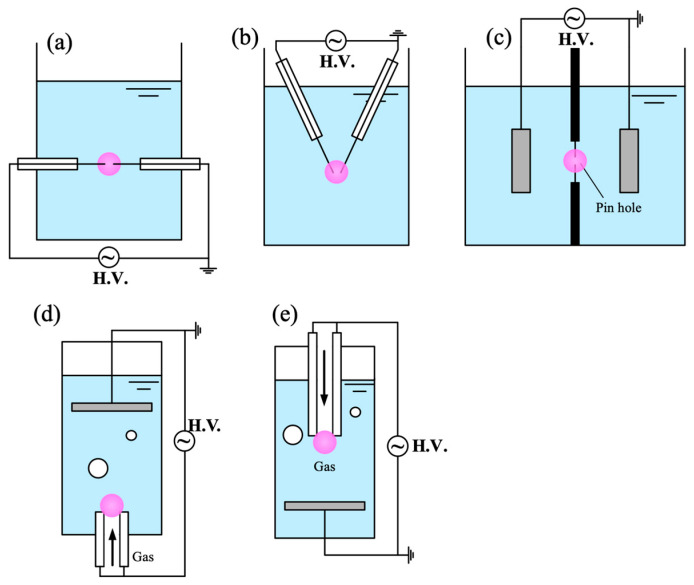
Schematic of several types of in-liquid plasma setups for material science: (**a**,**b**) pin-to-pin; (**c**) diaphragm discharge; (**d**,**e**) plasma discharge in bubble.

Such discharges have been widely applied in nanoparticle synthesis and configurations are shown in Figure 4, but they are less suitable for improving the hydrophilicity of hydrophobic CMs [129]. This limitation arises because hydrophobic particles tend to remain at the liquid surface, reducing the transport efficiency of plasma-generated reactive species to the material. For carbon synthesis, plasma enables synthesis of carbon nanospheres, nanosheets, and hierarchical carbon nanoballs by tuning pulse frequency and precursor type [128,130]. However, scaling up the process remains challenging. Although multiple parallel electrodes can be employed to increase production volume, breakdown near the lower electrodes become increasingly difficult once large amounts of carbon powder accumulate. A more practical approach may involve combining the discharge system with a circulation loop and a filtration unit, allowing continuous removal of synthesized powders and more stable discharge operation. In addition to the direct discharge in liquid, there are some other methods to generate plasma below the liquid, including diaphragm discharge [131], plasma discharge in the bubble which generated by bubble generator [15], etc.

**(c) Hybrid/advanced systems**

**Figure 5:**
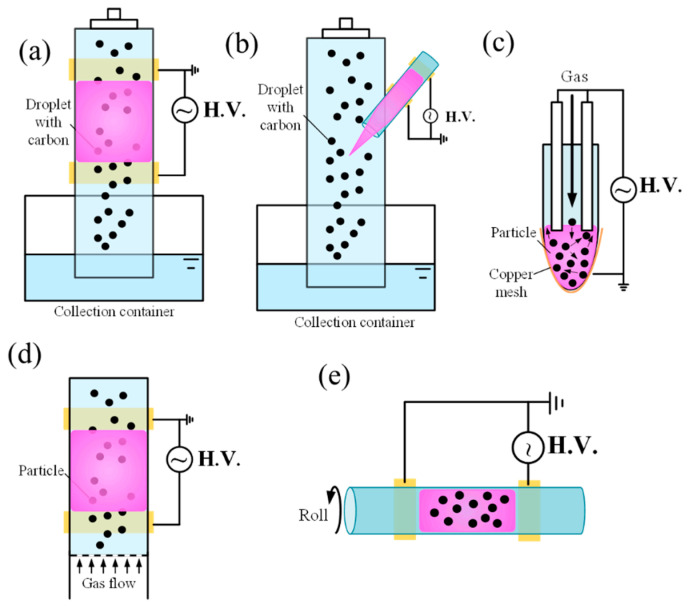
Schematic of several types of Hybrid/advanced systems for material science: (**a**) DBD reactor with water droplet; (**b**) APPJ with water droplet; (**c**,**d**) FB-DBD plasmas; (**e**) rotating-barrel DBD systems (The arrow indicates the direction of reactor rotation).

Hybrid plasma systems can be classified into two main categories: (i) gas discharges with a dispersed liquid phase, such as aerosol plasmas, and (ii) discharges in foams. In aerosol plasmas, liquid can be mixed with carbon powders and introduced into the discharge zone *via* a nozzle in the form of droplets (Figure 5). This approach significantly increases the plasma–liquid–solid contact area, thereby enhancing the efficiency of CM treatment. However, the process requires precise nozzle design and performance and is highly dependent on the particle size of the carbon powders [132]. In some cases, clogging or backpressure can occur, which limits operational stability. To date, some studies have reported carbon modification using discharges in foams, as this approach remains highly challenging and complex. The introduction of carbon into plasma-foam systems can alter the behavior of the liquid films between bubbles, influencing thinning and rupture processes, and thereby affecting the overall stability and lifetime of the foam.

In addition to these hybrid systems, several advanced plasma configurations have been developed for material processing, including rotating-barrel DBD plasmas (Figure 5e), fluidized-bed DBD plasmas (FB-DBD) (Figure 5c), and spark discharge APPJs (SDAPPJs). In conventional DBD plasmas, residual surface charges prevent transition into spark or arc regimes but can reduce the uniformity of filamentary discharges [133]. In rotating-barrel DBD systems, rotating electrodes improve uniformity by dispersing residual charges, while rotating dielectric plates further enhance control by simultaneously shifting both airflow and surface charge distribution [134]. The FB-DBD reactor operates by fluidizing catalyst powders with an upward gas flow while sustaining a plasma discharge through the particle bed. The motion of the particles stabilizes the plasma, increases plasma–substrate interactions, and prevents localized overheating. This configuration enables continuous, scalable, and efficient plasma-driven reactions, with demonstrated applications in CO_2_ conversion [135], methane reforming [135], ammonia synthesis [136], and nanocarbon production [137]. Compared to packed-bed DBD reactors, FB-DBDs provide superior mixing, more stable discharges, and true continuous operation [138].

### 2.2. Outlook on Reactor Design

Recent advances in APP technology have focused on scaling reactors for industrial CMs processing. Demonstrated approaches include rotating-barrel DBD reactors for uniform CNT treatment, multi-jet plasma arrays for roll-to-roll graphene functionalization, solution plasma systems capable of producing gram-to-kilogram quantities of CNTs dispersions within hours, and hybrid FB-DBD reactors for AC modification. A notable trend is the integration of in situ diagnostics into reactor design, enabling real-time monitoring of radical generation and optimization of functional group selectivity. APP reactor design evolves from simple laboratory-scale devices toward advanced, scalable platforms targeting applications, and environmental technologies. Looking ahead, several priorities can be identified. Firstly, development of circulator systems to bridge laboratory demonstrations with industrial production. Looking forward to integration of advanced diagnostics, closed-loop feedback, and AI/machine learning to figure out the relationship between surface functionalities with plasma parameters. The last, reducing energy consumption and by-products while enabling solvent-free or recyclable plasma-assisted processes. Like, advanced power modulation to improve energy efficiency, such as tailored pulse waveforms, exploitation of Penning effects in gas mixtures.

Regardless of configuration, several parameters critically determine the performance of APP reactors for CMs treatment. Electrode geometry and materials strongly influence discharge stability, uniformity, and contamination risk. Robust metals such as tungsten and stainless steel are commonly used, while in DBD systems ceramics offer long-term durability and quartz is favored when in situ diagnostics are required. Power supply characteristics also play a central role: the choice of pulsed, AC, or RF excitation defines whether the discharge operates in a filamentary or diffuse mode, with pulsed power generally providing greater control over energy deposition. Gas composition and flow rate dictate both the type and density of reactive species; noble gases such as helium promote stable diffuse discharges, whereas some other gases like O_2_, N_2_, NH_3_, CO_2_ enable heteroatom incorporation in the modification process. The plasma–material contact mode is equally important: direct plasmas promote strong etching and grafting, while remote plasmas minimize ion bombardment but rely more heavily on long-lived species. Finally, careful temperature management is essential to maintain non-equilibrium conditions compatible with thermally sensitive functional groups on CMs.

## 3. Progress of APP Technology on Material Science

As introduced in chapter I, plasma modification technology enables physical and chemical transformations on nanomaterial surfaces through interactions with chemically active species generated in gas-phase or plasma–liquid discharges. Different plasmas induced distinct surface chemistries, water plasma enhances the incorporation of aliphatic C–O (*sp*^3^) groups [139], reflecting its stronger reactivity and greater ability to disrupt graphitic structures compared with O_2_ plasma. Whereas CO_2_ plasma leads to more extensive surface oxidation, incorporating acidic groups such as –COOH, C=O, and –OH. N_2_ plasma treatment primarily introduces basic functional groups, such as –NH_2_, imines [140]. Notably, plasma-induced functionalization occurs rapidly, typically modifying only the uppermost monolayer. In this chapter, recent progress of APP technology in materials science will be introduced, with emphasis on treatments involving different gaseous and liquid plasmas.

### 3.1. Gas-Phase Plasma for Material Treatment

Gas-phase APP techniques can induce both physical alterations of carbon material texture and chemical functionalization depending on the plasma gas employed. The choice of gas is therefore critical in determining the modification pathway. O_2_-containing plasmas typically introduce OFGs while simultaneously increasing surface area. In contrast, N_2_ plasmas may deplete oxygenated surface sites and partial pore collapse [96]. Ar plasmas, though chemically inert, can still modify porosity and surface topology through ion bombardment, and such plasma modifications have proven effective in enhancing the adsorption, conductivity or other properties of CMs [141]. Here we discuss plasma treatments of carbon materials under various gas atmospheres and highlight how different plasma chemistries govern the resulting chemical transformations.

#### 3.1.1. O-Containing Plasma to Introduce Oxygen-Containing Groups

In plasma-based carbon treatment, O_2_ is one of the most widely employed gases for plasma-based modification of CMs, owing to its strong oxidative reactivity and versatility in generating OFG. Active species formed in the discharge, such as atomic oxygen and excited oxygen, react with carbon surfaces primarily through C=C bond cleavage, formation of C–O and C–OH intermediates, and subsequent rearrangement into more stable carbonyl (C=O) and –COOH [26,41].

Comparative studies highlight the distinct features of O_2_ plasma relative to wet chemical oxidation. Xia et al. [38] compared O_2_ plasma treatment with nitric acid oxidation, finding that acid treatment produced a wider variety of acidic groups including –COOH and –OH, whereas O_2_ plasma predominantly yielded C=O groups. Similarly, Huang et al. investigated O_2_ plasma versus nitric acid modification of viscose-based activated carbon fibers (VACFs) for use as vanadium oxide supports in low-temperature selective catalytic reduction (SCR). They reported that O_2_ plasma enhanced oxygen functionalities, improving catalyst dispersion and catalytic activity [39]. Treatments have also extended to bamboo-based activated carbon (BAC), where O_2_ plasma markedly altered surface chemistry by introducing C=O and –OH, thereby increasing acidity, polarity, and π–π/hydrogen-bonding interactions [73]. O_2_ plasma treatment of activated carbon fibers (ACFs) was shown to enhance water vapor adsorption, especially under low relative humidity, due to the generation of polar oxygen functionalities and unpaired carbon sites [142]. In the O_2_ DBD plasma regeneration of granular AC (GAC), Qu et al. speculate reactive species such as O_3_, •OH, •HO_2_, •O_2_^−^, and •RO were responsible for surface oxidation and renewal. Initially, plasma exposure increased surface area and pore volume due to mild activation; however, with successive cycles, micropore destruction and blockage occurred, decreasing both parameters. DBD plasma increased the population of –COOH while reducing basic surface functionalities, leading to a shift in overall acidity. Phenolic and lactonic groups fluctuated with cycle number, indicating complex oxidative modifications [143]. Similarly, atmospheric-pressure oxygen plasma treatment of ACFs increased the oxygen content from 7.8 at.% to 17.2 at.% after four treatment cycles, primarily introducing –OH and –COOH groups. However, the specific surface area and micropore volume decreased from 2121 to 1460 m^2^ g^−1^ and from 0.82 to 0.57 cm^3^ g^−1^, respectively, due to pore collapse and blockage [85]. Oxygen plasma also influences the textural and morphological properties of CMs. Plasma treatment was reported to slightly decrease particle size, total surface area, and micropore volume, while increasing meso/macropore surface area and average pore diameter. The surface fractal dimension decreased modestly, indicating mild structural smoothing [76]. These studies reveal that O_2_ plasma introduces two competing effects on carbon surfaces: the etching effect, which opens closed micropores, roughens surfaces, and increases surface area, and the grafting effect, which incorporates –COOH, C=O, and ester groups. Moderate treatments balance these effects to enhance surface reactivity and adsorption capacity, whereas excessive exposure or high discharge power can over-etch the structure, collapse micropores, and even degrade oxygen functional groups, leading to reduced oxygen content. Thus, careful optimization of plasma power, exposure time, and gas composition are essential to achieve uniform functionalization without structural degradation.

Beyond O_2_, other oxygen-containing gases have been employed for plasma treatment. CO plasma has proven advantageous for generating stable carbonyl functionalities while minimizing structural damage. CO plasma treatment achieved an O/C ratio as high as 0.69, significantly higher than those obtained with typical O_2_ plasma or low-pressure RF plasmas, which typically plateau around 0.1 [144]. Moreover, comparative studies of O_2_, CO_2_, and CO plasmas on cyanate ester-based composites confirmed that CO treatment not only provided the highest oxygen incorporation but also formed durable C=O groups rather than weakly bonded carbonate-like species [145].

#### 3.1.2. N-Containing Plasma to Introduce N-Containing Functional Group

Plasmas generated from NH_3_, N_2_, or NO_x_ gas are widely employed to introduce nitrogen-containing functional groups onto CM surfaces, thereby modifying their hydrophilicity, chemical reactivity, biological affinity, adsorption and catalytic performance. During plasma discharge, reactive nitrogen species (RNS) including NH•, NH_2_•, and N• radicals interact with defect sites and edge carbons, forming amine (–NH_2_), imine (–C=NH), and graphitic nitrogen functionalities.

Controlling the doping type and concentration is essential for optimizing the physical and chemical properties of plasma-treated CMs. Jung et al. [146] found that N_2_ ICP-plasma is especially effective due to its ability to introduce beneficial nitrogen species and enhance dispersion without compromising CNT integrity. N_2_ plasma treatment also has been applied to MOF-derived carbons, producing Co–N_x_ active sites and pyridinic-N functionalities that facilitate oxygen adsorption and enhance electron transfer kinetics in air battery cathodes [147]. DBD plasmas have been widely applied for scalable nitrogen functionalization. DBD treatment of MWCNTs enables surface modification for enzyme immobilization, producing stable interfaces with minimal damage to the carbon framework [78]. Ammonia plasma treatment has also been used to produce nitrogen-doped CNTs (NCNTs) that serve as metal-free electrocatalysts for CO_2_ reduction to formate in aqueous solutions, highlighting the catalytic potential of plasma-engineered nitrogen sites [148]. Air DBD plasma has also been used to introduce both oxygen and nitrogen functionalities onto electrospun lignin-based carbon nanofibers (LCNFs). Within just five minutes of treatment, reactive species (O^+^, O_2_^−^, O_2_^+^, N, N_2_^+^) introduced up to 15.24 wt% O and 11.48 wt% N, forming a range of groups such as C=O, –COOH, pyridinic-N, and pyrrolic-N. Enhanced wettability and polarity contributed to improved electrochemical performance [72].

#### 3.1.3. S-Containing Plasma to Introduce S-Containing Functional Group

Sulfur incorporation into CMs via plasma treatment has emerged as an efficient alternative to conventional high-temperature or acid-based sulfonation routes. In situ sulfur doping and surface functionalization can be achieved under mild, solvent-free conditions while maintaining the integrity of the carbon framework. Ma et al. [149] first synthesized sulfur-doped graphitic carbon nitride (g-C_3_N_4_) in in situ DBD plasma treatment under an H_2_S atmosphere. Compared with conventional thermal roasting, the plasma route achieved higher sulfur incorporation efficiency and preserved nanosheet morphology. In another study, O_2_/SO_2_ dual-doped PCs were prepared via non-thermal plasma treatment of CO_2_-pretreated biomass-derived carbon in flue-gas-relevant O_2_/SO_2_/N_2_ mixtures [150]. The simultaneous oxidation and sulfonation produced surface functionalities, significantly improving adsorption capacity of materials. Recently, da Silva et al. [151] use non-thermal plasma–assisted synthesis of sulfur-functionalized graphene was reported using an aqueous thiocyanate precursor. Within 1 h of treatment, covalently bound thiol (–SH) and sulfonate (–SO_3_^−^) groups were introduced without disrupting the *sp^2^* framework or crystallinity of graphene.

#### 3.1.4. C-Containing Plasma to Synthesize Carbon

Carbon-containing gases such as CH_4_, CH_3_OH, and CO_2_ are commonly used as plasma precursors for synthesizing carbon nanomaterials. While nanocarbon synthesis has traditionally been achieved through chemical vapor deposition (CVD) [152,153], solvothermal routes, sol–gel methods, laser ablation, etc., plasma-assisted processes provide distinct advantages, including solvent-free operation, fast processing, and tunable energy input.

In 2008, it has been demonstrated that non-thermal DC discharges sustained above water surfaces in CO_2_/N_2_/H_2_O gas mixtures can simultaneously drive CO_2_ decomposition and the formation of simple organic molecules under conditions analogous to prebiotic Earth chemistry [154]. The influence of plasma temperature on particle growth mechanisms was further elucidated in a study where the same plasma process produced diverse carbon nanoparticles [155]. At high temperatures, lateral growth led to organized textures such as acetylene-type CB or crumpled graphitic sheets, whereas lower temperatures favored radial growth and droplet-assisted condensation, producing amorphous, furnace-type CB. These results highlight the temperature-dependent competition between vapor condensation and surface growth during plasma-assisted carbonization.

Sun et al. [156] developed hybrid plasma configurations to combine thermal and non-thermal regimes for scalable carbon nanoparticle synthesis. A two-group arc discharge system integrating AC (non-thermal) and DC (thermal) discharges in a fluidized bed reactor enabled the production of CB and CNTs from propane. The AC discharge initiated propane decomposition into C_2_ radicals and H_2_, while the DC arc maintained the high-temperature carbonization necessary for CNT nucleation and growth. Subsequent heat treatment promoted controlled aggregation into nanostructured carbon. Furthermore, gliding arc plasma systems have demonstrated simultaneous syngas (H_2_ and CO) generation and carbon nanomaterials synthesis from CH_4_–CO_2_ mixtures [110]. Compared with DBD reactors, the gliding arc process produced a cleaner gas composition with narrower hydrocarbon distributions, while generating stable, conductive carbon nanoparticles and nanotubes as valuable by-products.

#### 3.1.5. Mixing Gas Plasma to Introducing Functional Groups

Air is widely used as a discharge gas because it is low cost, readily available, and yields a rich mixture of reactive species. However, its chemistry is complex and less well understood than that of pure O_2_ or N_2_ plasmas. Since air primarily consists of N_2_ and O_2_, with a volume ratio of approximately 3.76:1. It remains an open question whether the comparatively lower densities of reactive O and N species are as effective at introducing OFGs or nitrogen containing groups as in single-component plasmas. For CFRP-PEEK, air plasma improved surface polarity and hydrophilicity but lacked durable anchoring, while N_2_ plasma combined mechanical anchoring with stable hydrophilicity, yielding more persistent adhesion [157].

During air plasma treatment, both O and N functionalities can in principle form. Although nitrogen species are generally weaker oxidants, they may still interact with carbon materials leading to bond cleavage and the formation of nitrogen-containing surface sites. Okpalugo et al. [98] used ambient-air DBD to oxidatively functionalize CNTs and found optimal oxygen incorporation (~14.3 at.%) at ~0.5 kW, with improved thermal behavior. In the case of modify AC, air-based non-thermal plasma introduced C=O and ester groups and increased mercury removal despite a slight BET decrease; the O/C ratio rose to 14.5% with longer treatment [158]. Wang et al. [159] leveraged air plasma within an N,P,O co-doping strategy to enhance hydrophilicity, ion accessibility, and kinetics in carbon microspheres. Shen et al. [74] used spark-discharge APPJ (SDAPPJs) to modify AC, simultaneously improving porosity and surface chemistry. Liu et al. [160] employed air plasma treatment to enhance the interfacial adhesion between carbon fibers (CFs) and bismaleimide (BMI) resin. The interlaminar shear strength increased by 20.4%, reaching a maximum of 130.6 MPa after 900 s of plasma exposure. The improvement was primarily attributed to the introduction of polar OFG, which enhanced fiber surface polarity and chemical reactivity, while nitrogen incorporation remained limited. Naseh et al. [161] compared dry-air DBD with conventional acid treatments for MWCNTs. plasma avoided corrosives, was faster, and introduced more thermally stable groups (C=O/ether) than acids (predominantly –COOH) with less structural damage. Gliding arcs in air have also been explored. Du et al. [109] reported using gliding arc treat ACF increased oxygen content in ACF but with ~12% surface-area and ~31% pore-volume losses, and later raised ACF oxygen from 1.1% to 2.3% via –OH/–COOH incorporation [108].

In addition to the air plasma treatment, there are some other mixing gas discharges for the carbon modification. Mixed gases are often used to exploit Penning ionization, where a metastable species transfers energy to a second gas with lower ionization potential. The Penning effect not only enhances the overall ionization efficiency of the discharge, thereby facilitating plasma ignition and stabilization at lower voltages, but also enriches the reactive plasma chemistry by promoting the formation of additional ionic and radical species from the additive gas.

Sarra-Bournet et al. compared filamentary DBD (FDBD) and atmospheric-pressure Townsend discharge (APTD) for grafting –NH_2_ on PTFE. For high –NH_2_ density, FDBD in H_2_/N_2_ was optimal despite its aggressiveness. APTD in NH_3_/N_2_ preserved surface structure and offered safer, scalable processing [162], and compared favorably with low-pressure RF (RFGD) for biomedical PTFE functionalization [163]. Ar–NH_3_ DBD proved especially effective for functionalize with –NH_2_ and nitrogen-containing functionalities via synergy between etching (via O•, OH• radicals) and reactive NH• species promoted higher surface reactivity. López-Santos et al. [84] compared low-pressure MW and atmospheric pressure DBD plasmas for functionalizing diamond-like carbon (DLC) with –NH_2_. Among various conditions, Ar–NH_3_ DBD yielded the highest nitrogen incorporation (~10.6 at.%) and most effective activation, outperforming MW and N_2_-only plasmas, though nitrogen functionalities gradually decayed over time. Sequential or multi-stage processes further improve nitrogen functionalization efficiency. Moreover, NO-containing plasmas (Ar + NO or N_2_ + NO) have been shown to introduce pyridinic, pyrrolic, quaternary, and oxidized nitrogen species on graphite, demonstrating plasma’s ability to transform and immobilize nitrogen oxides on carbon frameworks [139].

In a two-step APPJ approach, initial Ar plasma activation generated surface dangling bonds on CNT arrays, followed by Ar/NH_3_ plasma treatment that grafted –NH_2_ via NH/NH_2_ radicals, demonstrating the necessity of combining surface activation and post-functionalization stages [164]. Similarly, a two-step DBD process using He plasma pre-treatment and subsequent NH_3_ chemisorption introduced amine, amide, and lactam species on MWCNTs. Plasma-induced defect sites and oxygen groups enhanced NH_3_ dissociation, enabling pyridinic-type nitrogen incorporation at energetically favorable defect positions [165].

Helium-assisted DBD reactors offer a significant advantage for carbon surface modification because they can generate spatially uniform plasmas even under atmospheric pressure. When a small amount of oxygen is introduced, He/O_2_ mixtures produce more homogeneous and controllable surface oxidation than pure O_2_ discharges, which typically exhibit filamentary streamers and require higher breakdown voltages. Consequently, He-based DBD plasmas offer significant advantages for the controlled oxidation of AC. For instance, DBD treatment using a He/O_2_ mixture increased the O/C atomic ratio from 0.22 to 0.48 after 30 min, primarily due to the enrichment of –COOH and C=O [166]. A similar approach using O_2_/He plasma was shown to introduce –OH, C=O, –COOH functionalities onto CNT surfaces, increasing the O/C ratio from 0.02 to 0.08 and thereby improving CNT–epoxy interfacial bonding [75]. Symmetric plasma exposure produced more uniform and abundant functional groups compared with asymmetric modes, while the water contact angle decreased from 104° to 50°.

Plasma post-treatment also serves as a versatile strategy for engineering the surface chemistry and morphology of carbon nanowalls (CNWs). By varying the gas composition, CNWs can be tuned from super hydrophilic (N_2_, O_2_, or H_2_ plasmas) to superhydrophobic (SF_6_ plasma), while maintaining their graphitic framework [167]. The morphological evolution depends strongly on the plasma chemistry: H_2_ and O_2_ plasmas promote edge etching, wall thinning, and branching; N_2_ plasma induces mild thinning without severe damage; C_2_H_2_F_4_ and SF_6_ plasmas deposit conformal fluorocarbon films, smoothing the surface and introducing C–F and –CF_3_ functionalities. Aldehydes introduced under Ar/H_2_ plasma, peroxides and C=O under Ar/O_2_ plasma, C–N (pyridinic-like) modes under Ar/N_2_ plasma, and C–F species under fluorocarbon-based plasmas. In DBD plasma-assisted amine functionalization, combining helium plasma activation with ammonia chemisorption at 200 °C effectively grafts –NH_2_ groups onto CNT surfaces, yielding materials with excellent aqueous dispersibility and biocompatibility [97]. Such hybrid plasma–gas chemistries highlight the potential of He-based discharges as gentle yet tunable platforms for multi-element surface modification.

Beyond surface activation, high-temperature plasma systems are increasingly utilized for carbon nanoparticle synthesis from hydrocarbon precursors. In an AC arc plasma process, methane decomposition at around 5000 K yielded both CNTs and spherical carbon nanoparticles (CNPs) through vapor condensation and catalytic growth. Fe electrodes promoted the formation of long, straight CNTs, while Cu and W electrodes favored spherical particles. Argon flow rate and methane concentration strongly influenced particle size, where higher Ar flow shortened residence time, producing smaller particles. Increasing current raised plasma temperature, enhancing graphitization and promoting more ordered carbon structures. APP systems can also enable direct growth of PCs on metal substrates. Using ethanol vapor and Ar as precursors, DBD plasma synthesis produced nest-like PCs with ~100 nm pores [44]. Electrode material significantly influenced morphology: Cu, Fe, Ag, and Pt supported well-formed porous structures, while Al and Au led to aggregated carbon deposits. Similarly, magnetically stabilized gliding arc discharge systems have been used for methane decomposition to produce diverse carbon nanostructures [111]. Here, the choice of buffer gas critically affected product morphology: CH_4_ and Ar atmospheres generated mixtures of spherical carbon nanoparticles and graphene nanoflakes (GNFs), while He and H_2_ atmospheres favored few-layer GNFs with high crystallinity, large BET surface areas, and excellent thermal stability.

Mixed-gas plasmas provide flexible routes for introducing heteroatoms and tuning surface chemistry in CMs. Air plasmas excel in oxidation and hydrophilicity enhancement, while Penning-assisted and He-based systems enable more selective, uniform, and energy-efficient functionalization. Together, these approaches underline the versatility of atmospheric-pressure plasma technologies on carbon processing.

**Figure 6:**
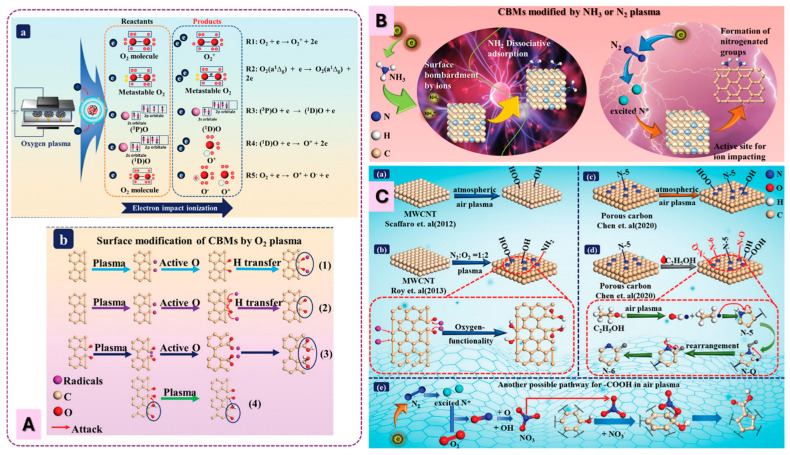
(**A**): Mechanism for introduction of OFGs on CM surfaces by O_2_ plasma. (**A**)/(**a**) Generation of active species. (**A**)/(**b**) Mechanism of OFGs generation by active species in plasmas. (**B**): Mechanism of typical N-containing plasmas for the introduction of NH_2_ on carbon surfaces. (**C**): Mechanism of typical N_2_&O_2_ plasmas for the introduction of functional groups on carbon surfaces. (**C**)/(**a**) Only OFGs are introduced on MWCNTs by air plasma. (**C**)/(**b**) N-containing groups introduced on MWCNTs by N_2_&O_2_ plasma treatment. (**C**)/(**c**,**d**) Schematic illustration of oxidation/nitrogenation of carbon surfaces. (**C**)/(**e**) Possible pathway for generation of COOH in air plasmas. Adapted from [41] with permission from John Wiley & Sons.

Sun et al. (Figure 6) summarizes the mechanisms proposed for the introduction of oxygen- and nitrogen-containing functional groups onto carbon materials under various plasma environments. Although the discussion focuses on low-pressure plasmas, the fundamental reaction pathways are largely applicable to atmospheric-pressure plasmas. However, low-pressure plasmas exhibit longer mean free paths, higher electron energies, cleaner gas-phase compositions, and reduced collisional quenching. Consequently, they typically generate higher densities of reactive oxygen species (ROS), stronger VUV radiation, and more spatially uniform glow discharges compared to APP systems. In Figure 6A, atomic oxygen and other oxygen radicals produced in O_2_ plasmas attack the carbon surface through hydrogen abstraction, radical addition, and oxidation steps, ultimately leading to the formation of OFGs such as –OH, C=O, and –COOH. Figure 6B illustrates that both N_2_ and NH_3_ plasmas generate reactive nitrogen species that can graft amine groups or other nitrogen functionalities onto carbon surfaces. Figure 6C highlights the more complex chemistry occurring in air plasmas, where mixed N_2_/O_2_ discharges can introduce either purely oxygenated groups or a combination of O- and N-containing functionalities, depending on plasma conditions and reaction pathways. Pathways for –COOH formation, NOx participation, and competitive oxidation–nitrogenation reactions are also shown. Overall, these mechanisms demonstrate how different gas chemistries steer the formation of specific surface functionalities, providing a foundation for tailoring carbon surfaces through both low-pressure and atmospheric-pressure plasma processes.

According to the literature, oxygen functionalization tends to be significantly more efficient than nitrogen incorporation, with O-content increasing up to 40 at.% but N-content typically below 10 at.% (Table 4). Energy-cost analysis further shows that optimized APP systems still exhibit the lower efficiency compared to low pressure plasma systems (5–20 MJ/mol) [38,168], whereas DBD-based oxidation can exceed 100 MJ/mol due to limited penetration. Nitrogen incorporation is strongly plasma-chemistry dependent, NH_3_/Ar mixtures yield the highest increase N (up to +7.9 at.%).

Despite these trends, direct comparison across plasma systems remains challenging. Many studies do not report key parameters such as treated mass, plasma energy consumption, discharge power density, or reactor efficiency, making normalization across devices difficult. Additionally, the intrinsic properties of carbon materials, including surface area, porosity, crystallinity, and defect density, substantially influence both the extent and nature of plasma-induced functionalization. Although APP systems often show lower energy efficiency than their low-pressure counterparts, they offer practical advantages such as eliminating vacuum infrastructure, reducing operational energy consumption, and enabling straightforward scale-up, making them highly attractive for industrial processing.

### 3.2. Gas–Liquid Interfacial Plasma for Material Treatment

Similarly to gas-phase plasmas, plasma–liquid systems can be categorized according to their generation methods and discharge configurations. However, a defining characteristic of these systems is the mode of interaction between the plasma and the liquid, which strongly determines plasma behavior, chemical reactivity, and material processing outcomes. To provide a systematic understanding, this chapter separates inorganic liquid plasma treatment (e.g., water- and aqueous-solution-based systems) from organic liquid plasma treatment (e.g., hydrocarbon or alcohol-based precursors), since the underlying mechanisms and dominant reactive pathways differ significantly between these environments.

#### 3.2.1. Inorganic Liquid Plasma Treatment

Water- and solution-based plasmas have emerged as versatile platforms for in situ oxidation, doping, and surface modification of CMs. Reactive species such as •OH, O, and O_3_ generated in gas–liquid interfaces enable efficient introduction of OFGs and sulfur-containing functional groups while operating under mild, solvent-compatible conditions.

Zhang et al. [169] utilized pulsed O_2_ plasma generated within H_2_O bubbles to oxidize CNTs in a gas–liquid hybrid discharge reactor. •OH produced in situ attacked C=C bonds and defect sites, introducing –OH, –COOH, and C=O on CNT surfaces. Kolacyak et al. [104] compared APPJ oxidation of CNTs under spray injection versus dry feeding. Spray-assisted treatment efficiently incorporated up to 6.9 at.% O while preserving CNT morphology, as water droplets provided cooling and prevented structural damage. In contrast, dry feeding caused higher defect formation. Ionita et al. [170] applied submerged Ar/O_2_ plasma jet treatment for carbon nanowalls (CNWs), transforming them into multilayer graphene-like flakes with improved dispersibility. The O/C ratio increased from 14% to 55%, corresponding to the formation of OFG and enhanced surface wrinkling while preserving graphitic domains. Hoshino et al. [171] used a needle-to-solution plasma system for graphite hydrophilization, achieving more energy-efficient OH radical generation than traditional SPP. Functionalization with –OH and –COOH enhanced dispersibility, particularly under multi-electrode configurations that improved radical generation at the liquid surface.

Plasma oxidation has also been extended to carbonaceous solids such as biochar (BC) and AC. DBD plasma treatment of BC in O_2_, H_2_O, and mixed atmospheres increased oxygen functionality (O/C ratio increase from 0.20 to 0.35) through reactions involving O, O_3_, and •OH radicals. O_2_ plasma caused micropore destruction and mesopore formation, while H_2_O plasma preserved microporosity; the O_2_ with H_2_O mixture provided balanced oxidation with minimal structural loss [172]. Similarly, H_2_O-DBD plasma modification of coconut-shell-based AC selectively introduced C=O groups, enhancing surface polarity and wettability without damaging the pore structure [173].

Beyond oxidation by pure water, solution plasmas can enable heteroatom doping. Silva et al. [174] demonstrated simultaneous sulfur functionalization and aqueous dispersion of graphene using plasma over potassium thiocyanate (KSCN) solutions, incorporating –SO_3_H and –SH groups. CMs can be sulfonated by plasma interact with 1 M H_2_SO_4_ solution, making a high-performance catalysts with high density of –SO_3_H groups [81,82,175]. Kharisova et al. [176] developed a liquid-phase micro plasma for nitrogen and sulfur co-functionalization of technical carbon using ammonium nitrate and sulfuric acid solutions (1 M H_2_SO_4_). The process efficiently introduced pyrrolic-N, graphitic-N, N–O, C–S, and –SO_3_H groups. Shirafuji et al. [177] functionalized MWCNTs in aqueous ammonia via solution plasma. Within two hours treatment, treated CNTs became uniformly dispersible in water, indicating successful incorporation of hydrophilic groups such as NO_2_, OH, COH, and COOH, although direct –NH_2_ attachment was not observed. Wettability control using plasma is further illustrated by APPJ irradiation of SWCNT films [178]. Plasma exposure can make CMs improve its super hydrophilic, while waterproof spray treatment rendered the surface superhydrophobic. Combined plasma–spray treatment achieved an intermediate contact angle of ~50°, confirming that surface energy tuning via plasma–chemical modification can be achieved with high precision.

**Figure 7:**
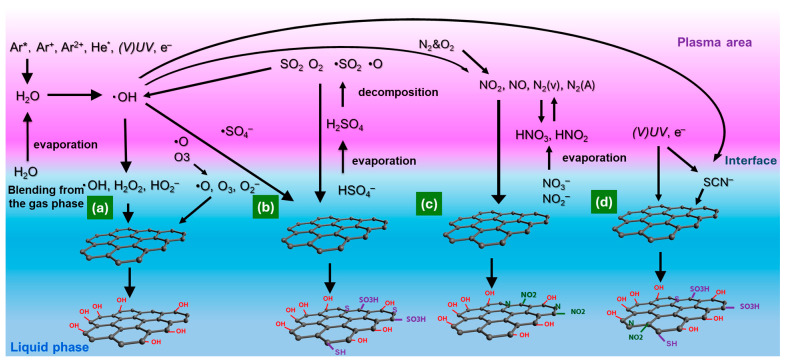
Mechanism of functional group introduction by plasma liquid interaction (He*: Metastable helium). (**a**) OFGs introduction on carbon surface by OH radicals in plasma discharge with purified water; (**b**) S-containing group introduction by plasma discharge with H_2_SO_4_ solution; (**c**) N-containing group introduction by plasma discharge with HNO_3_ solution; (**d**) N- and S-containing group introduction by plasma discharge with KSCN solution.

Figure 7 illustrates the mechanisms by which various functional groups are introduced onto carbon surfaces through plasma–liquid interactions. In Figure 7a, OH radicals, produced via water dissociation in plasma discharge and subsequent reactions, play a dominant role in oxidizing carbon surfaces due to their high oxidation potential. These species readily attack carbon bonds, generating oxygen-containing functional groups such as –OH, C=O, and –COOH. Beyond pure water systems, introducing specific electrolytes enables the formation of additional reactive species. In Figure 7b, discharges over H_2_SO_4_ solutions generate related sulfur–oxygen intermediates that can graft sulfonic, thiol, and other S-containing groups onto carbon surfaces. In Figure 7c, plasma treatment of HNO_3_ solution produces nitrogen oxides, which subsequently react at the carbon interface to form N-containing functional groups. Figure 7d shows that KSCN-containing solutions introduce both nitrogen- and sulfur-based radicals under plasma exposure, enabling simultaneous incorporation of N- and S-functional groups. Overall, these pathways demonstrate that combining plasma with different liquid chemistries provides a versatile platform for tailoring carbon surface functionalities, offering controllable routes to oxygenated, nitrogenated, sulfurized, or co-doped carbon materials.

Overall, plasma–liquid systems provide a unique, green, and tunable platform for carbon functionalization. They enable simultaneous synthesis and surface modification under ambient or near-ambient conditions without harsh chemicals or high temperatures. plasma–liquid processes can control the type and density of surface functional groups, while maintaining structural integrity.

#### 3.2.2. Organic Liquid Plasma Treatment

Early developments (2010s) demonstrated the potential of plasma processes for simultaneous carbon synthesis and surface modification. Jiang et al. developed a submerged-arc helium plasma reactor for the synthesis and in situ amination of carbon nanoparticles. Carbon particles were generated in benzene under plasma discharge and immediately exposed to ethylenediamine, allowing direct grafting of primary –NH_2_ via free-radical reactions. The resulting nanoparticles contained ~4.4 at% nitrogen contents, corresponding to amine or amide bonding states [179]. Kang et al. [180] employed the solution plasma process (SPP) to simultaneously generate mesoporous carbon nanoballs (CNBs) from benzene and deposit noble metal nanoparticles (Au, Pt) via electrode sputtering. The resulting CNBs exhibited uniform spherical morphology and well-developed meso/macroporous structures, demonstrating that SPP enables one-step, solvent-free fabrication of carbon–metal nanocomposites with controlled morphology and high surface functionality. In a related study, they synthesized structure-controlled carbon nanospheres (CNSs) from benzene by tuning the pulse frequency of the bipolar power supply. Glow discharge yielded amorphous carbon with high surface area and mesoporosity, while spark discharge produced short-range ordered graphene layers with turbostratic graphite structure [130]. Building on these advances, Ishizaki et al. [181] synthesized oxygen-containing nanocarbon materials via SPP using benzene and 1,4-dioxane mixtures. The resulting nanocarbons exhibited a turbostratic structure, with higher 1,4-dioxane content increasing oxygen incorporation (O/C ratio from 0.044 to 0.227) and defect density, and formed functional groups shifted from hydroxyl/epoxide to carbonyl and carboxyl species with the oxygen content increased.

Kim et al. [182] synthesized tungsten carbide nanoparticles uniformly dispersed on carbon or N-doped carbon matrices via SPP, with C–N, C=N, and CN groups identified. Similarly, SPP was applied to tune nitrogen configurations in N-doped carbon (N–C) materials. The precursor structure and additives strongly influenced bonding states: pyridine precursors favored pyridinic-N (45–50%), acrylonitrile yielded dominant –NH_2_-N (~49%), and anthracene additives increased graphitic-N content by 30–40% [183]. Boron-doped carbon nanoparticles were synthesized via SPP using benzene and triphenyl borate, incorporating ~0.67 at% B in forms such as BC_3_, B_4_C, and oxide [184].

He et al. [45] demonstrated a DBD-assisted one-pot synthesis of CQDs using N,N-dimethylformamide (DMF) as the sole precursor. This process produced CQDs with a disordered graphitic structure, surface –COOH/–OH groups for excellent water dispersibility, and bright blue fluorescence without external heating or chemical additives. Aromatic precursors facilitated nanocarbon growth through resonance-stabilized intermediates, with ring molecules reacting mainly at the plasma–solution interface to form polycyclic aromatic hydrocarbons, whereas linear molecules decomposed into small olefins and C_2_ species in the plasma core.

Morishita et al. systematically compared hydrocarbon precursors (hexane, hexadecane, cyclohexane, benzene) to elucidate nanocarbon formation mechanisms in SPP. The synthesis rate depended strongly on precursor structure: benzene yielded the highest rate, while linear alkanes produced much less. Aromatic precursors facilitated nanocarbon growth through resonance-stabilized intermediates, with ring molecules reacting mainly at the plasma–solution interface to form polycyclic aromatic hydrocarbons, whereas linear molecules decomposed into small olefins and C_2_ species in the plasma core [185].

Chung et al. [186] demonstrated liquid-phase plasma decomposition of hydrocarbons (hexane, benzene) for co-production of high-purity hydrogen and carbon nanomaterials. Only H_2_ and carbon nanoparticles were generated, achieving high yields of H_2_ [180 L (g·h)^−1^] and carbon [1% (g·h)^−1^] with no detectable CO_2_ emission. Choi et al. [127] further employed plasma cracking of benzene to simultaneously produce H_2_ and CB nanoparticles. The discharge generated primary CB particles (20–50 nm) that aggregated into clusters (400–500 nm). Post-annealing at 2500 °C increased crystallinity, forming multi-graphene shells with pentagonal and hexagonal faceting.

Table 5 provides an overview of major synthesis methods for carbon nanomaterials, comparing plasma-based and conventional techniques in terms of their precursors, advantages, and limitations. Plasma techniques, including SPP, submerged arc, gliding arc, and plasma cracking, offer one-step synthesis, in situ heteroatom doping, and environmentally friendly operation, but often face challenges such as limited throughput or size control. Traditional methods like CVD, arc discharge, and laser ablation provide high crystallinity and established scalability but require high temperatures or expensive setups. Overall, the table highlights the complementary nature of plasma and conventional synthesis routes, each offering distinct benefits depending on the desired nanocarbon structure and application.

### 3.3. Application of the APP Treated Materials

APP-treated CMs have demonstrated remarkable potential in energy, environmental, and biomedical applications, where surface-controlled functionalities govern catalytic, adsorption, and biocompatible behaviors. The following sections summarize recent advances in these application domains, emphasizing the structure–property relationships and processing strategies that link plasma conditions to material performance.

#### 3.3.1. Applications in the Energy Field

Since the first report of N-doped CNT arrays as oxygen reduction reaction (ORR) electrocatalysts in 2009 [190], heteroatom-doped carbons have attracted great interest as metal-free catalysts due to their high activity, durability, and fuel selectivity. Recent advances highlight the versatility of plasma-based heteroatom doping across different energy applications. Doping alters the charge or spin density of carbon atoms, promoting oxygen adsorption, weakening O–O bonds, and enhancing ORR performance. Among various strategies, plasma-assisted techniques, particularly SPP and DBD, offer efficient, one-step, and solvent-free routes for in situ heteroatom doping [42,191].

Recent studies demonstrate the broad applicability of plasma doping across energy-conversion and storage systems. DBD plasmas have been employed to fabricate bifunctional ORR/OER electrocatalysts for fuel cells and metal–air batteries [192], and to prepare carbon cathodes for Zn-ion hybrid supercapacitors, achieving specific capacitances up to 215 F g^−1^ and energy densities of 54 Wh kg^−1^ with nearly 100% capacity retention after 10,000 cycles [159]. Air DBD treatment further enabled O/N co-doping of carbon nanofibers, significantly improving conductivity and rate capability, with capacitance retention exceeding 100% after 2000 cycles due to enhanced wettability [72]. RF N_2_ plasma modification of MOF-derived N–CNT composites yielded efficient sodium–air battery cathodes with low overpotential (0.35 V) and >80% round-trip efficiency after 150 cycles [147].

At larger scales, direct plasma fluorination of GO has been demonstrated at ~9 kg h^−1^, producing F-doped materials for high-performance supercapacitors with energy densities of 25.9 Wh kg^−1^ and stable cycling over 20,000 cycles [193]. Plasma-assisted defect engineering further enhances catalytic activity; for example, plasma-etched S-doped graphene reduced hydrogen evolution reaction (HER) overpotential from >700 mV to 178 mV at 10 mA cm^−2^ [86]. Two-step (He + NH_3_) DBD treatment enables selective nitrogen functionalization of MWCNTs without structural degradation, improving conductivity by approximately 11 orders of magnitude [165]. Conductive carbons produced by plasma cracking of benzene show conductivities up to 16.8 S cm^−1^ after annealing, outperforming commercial Super-P and maintaining stable Li-ion battery cycling for over 80 cycles with minimal capacity loss [127].

SPP provides a flexible approach for synthesizing doped and composite carbons. O-rich nanocarbons synthesized from benzene–dioxane mixtures *via* SPP exhibit stable ORR performance and strong methanol tolerance [181]. Mesoporous carbon nanoballs decorated with Au/Pt nanoparticles demonstrate high ORR activity suitable for Li–O_2_ batteries [180], while WC/N–C nanocomposites show enhanced ORR onset potential (–0.29 V vs. –0.36 V for WC/C) and stable current retention (87% after 20,000 s) [182]. N-doped carbons prepared by SPP achieve ORR onset potentials up to 0.76 V (vs. RHE) with near four-electron transfer behavior [183]. Boron-doped carbon nanoparticles synthesized via SPP exhibited a positive shift in ORR onset potential (~20 mV), higher current density, and a partial transition toward a four-electron reduction pathway, confirming the beneficial role of boron incorporation [184]. Plasma treatment in solution has also been explored for heteroatom doping: N-doping in ammonium nitrate solution enhanced the ORR onset potential by +0.25 V, while S-doping in sulfuric acid induced a larger +0.31 V shift and higher limiting current (6.2 mA cm^−2^), demonstrating the tunable electronic effects of plasma-assisted heteroatom incorporation [176].

Conductive carbons produced by plasma cracking of benzene show conductivities up to 16.8 S cm^−1^ after annealing, outperforming commercial Super-P and maintaining stable Li-ion battery cycling for over 80 cycles with minimal capacity loss [106]. In thermoelectric systems, APPJ treated of CNT films enhances their surface wettability, increasing the output voltage of water-floating CNT thermoelectric generators by 30% under sunlight (1 kW m^−2^) with wind (3 m s^−1^) conditions [178].

Plasma etching has also been employed to engineer active defects in doped CMs. For example, a plasma-etching strategy applied to S-doped graphene significantly enhanced its hydrogen evolution reaction (HER) activity, where defect density effectively increased active sites without compromising conductivity or dopant stability [194].

Overall, plasma-based heteroatom doping and nanocarbon synthesis combine precision, scalability, and multifunctionality, enabling the development of next-generation CMs for energy conversion, storage and catalysts.

#### 3.3.2. Applications in the Environmental Field

Industrial effluents release a wide range of hazardous contaminants posing severe ecological and human health risks, like pharmaceuticals, dyes, heavy metals, herbicides, and pesticides. These emerging pollutants accumulate in aquatic systems and can cause antibiotic resistance, endocrine disruption, and carcinogenic effects. Pharmaceuticals such as antibiotics, hormones, and antidepressants are particularly concerning due to their persistence in water cycles. Heavy metals are non-degradable and bioaccumulative, while synthetic dyes from textiles, cosmetic, and paper industries often resist biodegradation. Similarly, herbicides and pesticides leach from agricultural soil into groundwater, leading to chronic toxicity, thyroid dysfunction, and developmental disorders.

Adsorption is an effective method for removing pharmaceutical pollutants from water, with surface-modified carbon showing excellent performance due to its tunable textural and chemical properties. Adsorption occurs as a result of surface energy minimization, involving multiple interactions such as hydrogen bonding, electrostatic attraction, π–π stacking, and dipole–dipole forces [195,196]. Van der Waals interactions and hydrophobic effects also play roles. In particular, hydrophobic interactions between nonpolar groups facilitate the binding of organic contaminants, though they are non-specific and arise mainly from entropy gain as solutes leave the aqueous phase [197].

Surface modification strongly influences the adsorption capacity of CMs. Plasma or ozone treatments can alter pore structure, surface roughness, and chemical composition, incorporating OFG such as phenolic or carboxylic acids that enhance adsorption via hydrogen bonding at low pH and π–π interactions at high pH [76,198]. Also, the modification methods by plasma and ozone seriously impact the pore size distribution and topography of ACs [141]. Sulfur doping introduces thiol (–SH) and sulfonic (–SO_3_H) groups that bind strongly to heavy metals through Lewis acid–base interactions to enhance the adsorption performance by forming strong interactions with heavy metals [199], while nitrogen- and phosphorus-doped carbons provide additional sites for hydrogen bonding and electron donation, facilitating the capture of pharmaceutical and metal ions [197].

Gas composition strongly affects performance: O_2_ plasma treatment increased surface area and introduced oxygen-containing groups, enhancing pentachlorophenol (PCP) adsorption, whereas N_2_ plasma treated CMs showed reduced PCP adsorption due to reduced surface area and depleted oxygen functionalities [96]. Helium–oxygen DBD plasma enhanced the ion adsorption capacity of AC by introducing –COOH and C=O, without significant pore damage [75]. DBD plasma has also been applied for regeneration of GAC. Although plasma treatment restored surface area after initial cycles, full adsorption recovery was limited by pore blockage, increased carboxylic groups weakening π–π interactions with AO7 and by-product accumulation [143]. Oxygen plasma treatment of bamboo-derived AC also enhanced aniline adsorption via π–π and hydrogen bonding interactions [73]. O_2_ with H_2_O DBD plasma-modified biochar achieved more than 10 times higher Hg^0^ adsorption (36.7–269.4 μg g^−1^) than raw biochar (3.7 μg g^−1^), outperforming commercial ACs [172]. Similarly, air gliding-arc plasma introduced oxygen functionalities on AC that increased Pb^2+^ and Fe^2+^ uptake markedly [109] and improved Acid Orange II dye removal by ~21% after 20 min modification, emphasizing that surface chemistry (introduce oxygen groups), rather than total surface area, dominates adsorption efficiency [108]. Similarly, oxygen plasma treatment enhanced HCl gas adsorption on ACFs by up to 300%, though excessive cycles led to pore damage and decreased performance. Ar plasma treatment decreased HCl removal, confirming the key role of oxygen groups [85]. Optimization of plasma parameters is essential: short or moderate treatments promote micropore opening, surface roughness, increase BET surface area, and functional group incorporation to improve adsorption capacity and accessibility of active sites, whereas excessive power or duration causes pore collapse, reduce overall surface area, and loss of activity. Balancing structural integrity with chemical activation is thus critical for maximizing adsorption efficiency [200].

Plasma treatment of PCs in flue gas environments also generates highly active sites for Hg^0^ capture without chemical additives. Functional group analysis showed the activity sequence for Hg^0^ chemisorption as C–SO_2_ > C=O > C–S > C–SO > C–SO_3_, indicating that oxygenated sulfur species play dominant roles [150]. Air-based plasma treatment further improved mercury removal efficiency in AC, with performance recovery after re-functionalization confirming the essential role of oxygen functional groups. At 130 °C, ester groups exhibited stronger Hg^0^ adsorption energy than C=O, relevant to industrial flue gas conditions [158]. Gas plasmas containing NO or H_2_O have been used to functionalize carbons for selective NO removal, highlighting their potential in gas-phase environmental remediation [139].

Beyond adsorption, plasma-based modification enables photocatalysis and advanced oxidation. DBD-modified heteroatom-doped g-C_3_N_4_ showed enhanced visible-light activity for pollutant degradation [149]. APPJs treatment on CMs improving dye like methylene blue removal through simultaneous pore activation and oxygen functionalization [74]. In addition to contaminant removal, plasma-functionalized CMs also serve as efficient electrocatalysts for H_2_O_2_ generation in electro-Fenton systems. Pulsed O_2_ plasma oxidation of CNT cathodes increased H_2_O_2_ yield from 102 mg L^−1^ to 146 mg L^−1^ at −0.85 V, enabling 95% degradation of methyl orange compared to 40% with untreated CNTs. The improvement was attributed to enhanced oxygen accessibility and surface-active sites for O_2_ reduction [169].

#### 3.3.3. Applications in the Biomedical Field

Plasma-assisted modification of carbon nanomaterials has attracted increasing attention in biomedical applications owing to their ability to introduce bio-functional groups, enhance hydrophilicity, and improve biocompatibility under solvent-free and mild conditions. Non-thermal plasmas generate a mixture of energetic electrons, ions, and reactive neutral species capable of inducing selective surface reactions without significant bulk damage. These unique physicochemical environments allow precise control of nanocarbon surface functionalities and have been exploited for the synthesis and activation of carbon nanostructures intended for diagnostics, tissue regeneration, and therapeutic systems [201].

Plasma activation coupled with surface reactions such as Schiff-base chemistry enables the fabrication of hydrophilic and chemically active CNT surfaces, providing versatile platforms for biosensing, drug delivery, and bio-composite engineering where aqueous dispersibility and stable bio-functional interfaces are essential [202]. For biomedical surface functionalization, atmospheric-pressure discharge in NH_3_/N_2_ has been shown to provide sufficient amine incorporation while preserving the morphology of carbon substrates, offering a gentle and controllable alternative to low-pressure plasma systems [162,163]. Similarly, amine functionalization of MWCNTs can also be achieved through helium DBD plasma activation followed by ammonia chemisorption, yielding surfaces suitable for advanced biosensor applications [97]. In another approach, a two-step APPJ process has been employed to –NH_2_-functionalize CNT arrays with high spatial precision. The resulting patterned CNT architectures act as robust scaffolds for biomolecule immobilization in biosensor devices [164].

Beyond surface activation, plasma-based synthesis has opened new routes toward multifunctional CMs with inherent biomedical utility. Submerged-arc helium plasma enables a one-step formation of amine-functionalized carbon nanoparticles with high dispersibility and surface stability. These nanoparticles exhibit favorable biocompatibility and chemical robustness, making them promising candidates for controlled drug release, bioimaging, and vaccine carrier systems [179]. DBD plasma can produce CQDs with tunable fluorescence and abundant oxygen-containing groups, enabling fast and reagent-free detection of biologically relevant analytes such as H_2_O_2_, Fe^3+^, and glucose. When applied to human serum, the CQD probe exhibited quantitative accuracy comparable to standard clinical assays, with recoveries of 90–100% [45].

Table 6 summarizes how plasma-engineered carbon materials are applied across energy, environmental, and biomedical fields. Plasma enables rapid heteroatom doping, defect creation, and surface activation, which enhance catalytic activity, electrode conductivity, adsorption capacity, and biocompatibility. Different plasma processes introduce functional groups or structural changes that directly translate into improved performance in applications such as ORR/HER catalysis, batteries and supercapacitors, pollutant removal, sensing, and drug delivery. Overall, the table highlights plasma as a versatile tool for tuning the surface chemistry and functionality of carbon materials to meet diverse technological needs.

## 4. Current Challenges and Future Perspectives

Despite the significant progress of APP technology for CMs modification and synthesis, several challenges remain before its widespread adoption in industrial and advanced research applications.

### 4.1. Control of Functional Group Selectivity and Carbon Structure

A persistent challenge in plasma modification is the precise control over the type, density, and place of functional groups introduced onto carbon surfaces. While oxygen- or nitrogen-based discharges readily incorporate functional groups, the resulting functionalities are typically a heterogeneous mixture of –OH, C=O, –COOH, and other oxygen-containing groups. For instance, O_2_ plasmas tend to form C=O and –COOH, whereas CO plasmas yield more stable functionalities but with limited tunability [144]. Achieving the chemical selectivity and reproducibility comparable to wet-containing plasma, due to the non-uniform plasma discharge.

Advancing this control will require the coupling of computational plasma modeling (particle-in-cell and global kinetics models) with in situ diagnostics such as OES, absorption spectroscopy, and LIF. These tools can quantify transient reactive species and correlate their evolution with functional group formation, enabling predictive understanding of functional group pathways. Combined with machine learning-driven optimization could enable closed-loop control of plasma conditions. This would allow reactors to autonomously tune parameters such as voltage, pulse frequency, and gas flow to achieve target surface chemistries with high reproducibility. The balance between chemical activation and structural preservation is another critical consideration. Plasma exposure introduces lattice defects that facilitate covalent bonding but may also degrade mechanical integrity, electronic conductivity, and crystallinity, especially in delicate nanostructures such as SWCNT and graphene. Overexposure can cause excessive etching and graphitic layer destruction, while underexposure results in poor surface reactivity. Future work should emphasize fine-tuned plasma dosage control to maintain the intrinsic properties of CMs while achieving uniform functionalization.

Another unresolved issue concerns the stability of plasma-generated functionalities under ambient or process conditions. Certain OFGs are metastable and may desorb or decompose over time, diminishing long-term performance. To address this, post-treatment stabilization approaches should be developed to anchor functional groups while preserving desired electronic and wetting properties.

### 4.2. Reactor Design, Scale-Up, and Sustainability

While the principles of APP reactors are well established, translating laboratory configurations into industrial-scale systems presents substantial challenges. Current research typically focuses on small substrates or small amount powder batches, whereas industry applications require reactors capable of processing kilograms of CB, meters of fiber, or large-area films with uniform treatment. Recent innovations such as rotating-barrel DBDs, FB plasma reactors, and multi-APPJ array systems show promise for achieving uniform exposure and continuous operation. However, challenges persist in maintaining homogeneous discharge distribution, mitigating electrode erosion, preventing powder agglomeration, and controlling fouling during extended operation. Moreover, although APP eliminates vacuum requirements, the energy cost per functionalized mmol of surface groups remains high in many configurations, driven by inefficient power coupling, short residence times, and limited penetration depth.

For sustainability, APP is inherently greener than traditional acid oxidation, minimizing pollution. Nonetheless, the overall environmental footprint depends strongly on reactor design, power electronics, gas utilization, and heat management. Improvements may come from advanced power supplies, optimized synergistic gas mixtures, and coupling renewable or waste-heat energy sources.

To support industrial transition, APP systems must be evaluated using quantitative green metrics, including the E-factor, process mass intensity (PMI), and energy cost per mmol of functional groups. However, many existing studies do not provide these assessments, making cross-comparison difficult. A key issue is the lack of standardization in energy accounting. Some works calculate the total energy consumption of the entire process (including gas flows, power supplies, and auxiliary systems), whereas others consider only the electrical power delivered to the plasma discharge. Likewise, comparisons between plasma methods and conventional chemical treatments are often made using different materials or inconsistent conditions, further complicating objective benchmarking. A unified methodology will be essential for enabling fair evaluation and accelerating industrial adoption. Integration of waste-gas recovery, catalyst-assisted plasma chemistry, and low-cost electrode materials can further reduce both energy consumption and operating cost. Ultimately, achieving economically viable large-scale APP reactors will require a holistic design strategy that integrates mechanical, electrical, thermal, and plasma-chemical optimization.

### 4.3. Outlook

APP represents a green, versatile, and scalable tool for engineering the surface chemistry and morphology of CMs. Future progress will rely on synergistic advances in reactor engineering, plasma–surface reaction understanding, and digital process intelligence. As diagnostic tools, plasma modeling, and machine learning continue to mature, APP systems are expected to deliver the precision and reproducibility of conventional chemical synthesis while preserving their inherent environmental advantages.

Importantly, overcoming the current challenges in APP-based carbon modification requires strong interdisciplinary collaboration. Researchers in plasma physics and plasma chemistry primarily focus on discharge characteristics, reactive species formation, and improving the energy efficiency of plasma reactors. In contrast, materials scientists concentrate on structural integrity, functional group stability, and the broader performance metrics of modified carbon materials. Application-oriented researchers—working in catalysis, energy storage, environmental remediation, or biomedicine—contribute additional insights by defining performance requirements and identifying bottlenecks in real-world deployment. Coordinated collaboration among these communities will be essential to align plasma conditions with material-specific needs, integrate plasma processes with catalytic or post-treatment strategies, and accelerate the translation of APP-based modifications into practical technologies.

Looking forward, APP has the potential to emerge as a cornerstone technology for sustainable carbon nanomaterial manufacturing, enabling applications in catalysts, batteries, supercapacitors, filtration membranes, flexible electronics, and biomedical devices. The convergence of scalable reactor design, catalyst-coupled plasma chemistry, and real-time process control will define the next generation of APP-enabled carbon material processing.

## Figures and Tables

**Table 2:** Key plasma parameter range in different plasmas [71,72].

Discharge Type	*T_e_* (eV)	*T_g_* (K)	*n_e_* (m^−3^)	*E*/*N* (Td)	Power/Energy
DBD	2~3	300~600	10^19^–10^21^	100–500	<10 µJ per pulse
APPJ	1~2	300~600	10^19^–10^20^	100–500	<10 µJ per pulse
Glow discharge	1~2	300~1000	10^18^–10^19^	100–200	5–100 W
RF plasma	2~10	300~1000	10^15^–10^18^	50–300	50–500 W
Pulse spark discharge	1~2	500–3000	10^20^–10^24^	>1000	10–100 W
Gliding arc plasma	~1	2000~6000	10^20^–10^22^	<100	10–100 W
Thermal arc plasma	~1 (≈*T_g_*)	5000~10,000	10^21^–10^23^	<10	100–1000 W

**Table 3:** Classification of atmospheric pressure plasma systems for material treatment: configurations, Properties, and representative applications in carbon materials.

Category	Type of the Plasma	Properties (Advantages, Limitations)	Application on CMs
Gas-phase plasmas	RF plasma, microwave plasma, inductively coupled plasma (ICP), arc discharge, pulsed DC plasma	Uniform modification, controllable plasma chemistry, large-scale surface treatment; May require costly gases, risk of damage under high power	CNT purification by O_2_ plasma; N-doping of CNTs by NH_3_ plasma; S-doped porous carbon using O_2_/SO_2_ plasma, etc.
Plasma above liquid	DBD, APPJ, gliding arc discharge, corona discharge	compatible with continuous flow; Limited penetration depth, surface-localized modification only	Carbon wettability improvement; carbon modification
In-liquid plasmas	Streamer, arc discharge, spark discharge, micro-pulse discharge	Strong oxidation, direct nanoparticle synthesis in solution, no dispersants needed; Electrode erosion, solvent degradation, scaling challenges	CMs synthesis; CNT oxidation in water bubbles; in NH_3_ solution for MWCNTs functionalization
Hybrid/advanced systems	Rotating-barrel DBD, fluidized-bed plasmas, SDAPPJ, multi-APPJ arrays	High scalability, uniform treatment of powders/fibers, versatile functionalization; Still in development, energy efficiency and control challenges	Carbon modification by SDAPPJ; carbon synthesis in fluidized-bed plasma

**Table 4:** Summary of plasma carbon materials treatment performance.

	Plasma	Power [W]	Treatment Time [min]	Increasing O Contents	Energy Cost of O (MJ/mol)	Increasing N Contents	Energy Cost of N (MJ/mol)
Xia 2007 [38]	HNO3 treatment (Not plasma)	Not reported	Not reported	8.70 at.% (XPS)	N/A	1 at.% (XPS)	N/A
Huang 2021 [142]	O_2_ DBD (9 kV)	Not reported	15 min	37.14 at.% (XPS)	N/A	0	N/A
Wu 2012 ACF [73]	O_2_ DBD	100	16 min	0.24 mmol/g (titration)	150–200 MJ/mol	0	N/A
Qu 2009 [143]	Air DBD	100 W	180 min	0.143 mmol/g (titration)	~150 MJ/mol	Not reported	N/A
Naseh 2010 [161]	DBD Air	90 W	9 min	3.2 at.% (XPS)	N/A	Not reported	N/A
Zaldivar 2012 HOPG/GnP/CNT [148]	He/CO APPJ	Not reported	12–48 passes	39.8 at.% (XPS)	N/A	Not reported	N/A
Zaldivar 2012 CNT/polymer [145]	Ar/O_2_ APPJ	Not reported	3–48 passes	21 at.% (XPS)/11 at.% after rinse	N/A	21 at.% (XPS)/0% after rinse	N/A
Zaldivar 2012 CNT/polymer [145]	Ar/CO_2_ APPJ	Not reported	3–48 passes	18 at.% (XPS)/11 at.% after rinse	N/A	9 at.% (XPS)/1 at.% after rinse	N/A
Zaldivar 2012 CNT/polymer [145]	Ar/CO APPJ	Not reported	3–48 passes	31 at.% (XPS)/29 at.% after rinse	N/A	−4 at.% (XPS)	N/A
Park 2004 (ACFs) [85]	Ar/O_2_ (1%) RF	300 W	180 s (4 passes)	9.4 at.% (XPS)/0.38 mmol/g (titration)	~140 MJ/mol	0	N/A
López-Santos 2009 [84]	N_2_–DBD	50 W	1 h	5.2%(XPS)/2.1 at.% after 1 mouth	N/A	−0.8 at.% (XPS)/−2.3 at.% after 1 mouth	N/A
	Ar/NH_3_–DBD	50 W	1 h	–3.7 at.% (XPS)/–0.7 at.% after 1 mouth	N/A	7.9 at.% (XPS)/3.3 at.% after 1 mouth	N/A

N/A: The energy cost cannot be calculated because the necessary experimental conditions are not provided.

**Table 5:** Summary of synthesis methods for carbon nanomaterials and their key characteristics.

Synthesis Method	Typical CMs Produced	Precursor	Advantages	Limitations	References
Arc Discharge	CNTs	Graphite electrodes	High crystallinity	Purification required; mixture of species; energy intensive	Iijima et al.; Bethune et al. [21,22]
Solution Plasma Process	CNPs, CNBs, CNSs, N-doped Carbon, B-doped carbons, Graphitic Carbon Shells	Benzene, Benzene/dioxane, Pyridine, acrylonitrile, triphenyl borate; DMF	One-step synthesis; doping; rich OFGs	Small batch size; Liquid safety	Kang et al. [130,180]; Ishizaki et al. [181,183]; Kim et al. [182]
Submerged Arc in Liquid	CNPs	Aromatic liquids	One-step doping, liquid-phase control	Low throughput; broad size	Jiang et al. [179]
Liquid-phase Plasma Cracking	CB	Benzene, hexane	CO_2_-free H_2_; high purity	Post-annealing needed	Chung et al. [186], Choi et al. [127]
DBD assisted one-pot synthesis	CQDs	DMF	Low-temperature, additive-free	Limited crystallinity	He et al. [45]
Gliding Arc Plasma	CNPs, CNTs, amorphous carbon	CH_4_–CO_2_	Co-produces H_2_, CNMs	Needs tuning for uniformity	Tu et al. [110]; Wang et al. [111]
DC/AC Hybrid Arc (Fluidized Bed)	CB, CNTs	Propane	Scalable; hybrid thermal/NT	Requires powder circulation	Sun et al. [156]
Chemical Vapor Deposition	CNTs, CNFs, graphene	CH_4_, C_2_H_2_, CO	High crystallinity; scalable	Requires high temperatures; catalyst residues	[187,188]
Laser Ablation	CNTs, fullerenes	Graphite + catalyst	High purity product	Expensive, low yield	Scott et al. [189]

**Table 6:** Major Application Areas of Plasma-Engineered Carbon Materials.

Application	Relevant Carbon Material	Plasma Role	Representative Advantages
Energy Catalysis (ORR/OER/HER)	N-, S-, B-doped carbons; WC/N–C, Au/Pt–CNBs, MOF-derived N–CNTs	In situ doping, metal deposition	Higher catalytic activity, lower overpotential, improved durability
Energy Storage (Batteries, Supercapacitors)	CNTs, doped carbons, mesoporous carbons, fluorinated GO	Surface functionalization, doping, pore activation	Increased capacitance and energy density; improved conductivity; long cycle life
Environmental Purification (Adsorption of pollutants)	AC, biochar, S/N/P-doped carbons	Surface activation, oxygen functionalization and doping	Enhanced adsorption, catalytic degradation
Advanced oxidation & photocatalysis	Heteroatom-doped g-C_3_N_4_, O-rich CNTs	Oxygen functionalization	Enhanced photocatalytic degradation; higher H_2_O_2_ production in electro-Fenton systems
Biosensing & Bioimaging	Amine-CNTs, patterned CNTs, CQDs	Surface modification	Improved hydrophilicity and dispersibility, high sensitivity and stability in electrochemical and optical biosensors, fluorescent nanocarriers
Drug Delivery	O-rich CMs	Plasma-based surface passivation; One-step synthesis	Biocompatible, stable, potential for controlled drug release and vaccine carriers

## Data Availability

No new data were created or analyzed in this study. Data sharing is not applicable to this article.
